# Dengue Virus Targets the Adaptor Protein MITA to Subvert Host Innate Immunity

**DOI:** 10.1371/journal.ppat.1002780

**Published:** 2012-06-28

**Authors:** Chia-Yi Yu, Tsung-Hsien Chang, Jian-Jong Liang, Ruei-Lin Chiang, Yi-Ling Lee, Ching-Len Liao, Yi-Ling Lin

**Affiliations:** 1 Institute of Biomedical Sciences, Academia Sinica, Taipei, Taiwan; 2 Department of Medical Education and Research, Kaohsiung Veterans General Hospital, Kaohsiung, Taiwan; 3 Department of Microbiology and Immunology, National Defense Medical Center, Taipei, Taiwan; 4 Genomics Research Center, Academia Sinica, Taipei, Taiwan; Washington University School of Medicine, United States of America

## Abstract

Dengue is one of the most important arboviral diseases caused by infection of four serotypes of dengue virus (DEN). We found that activation of interferon regulatory factor 3 (IRF3) triggered by viral infection and by foreign DNA and RNA stimulation was blocked by DEN-encoded NS2B3 through a protease-dependent mechanism. The key adaptor protein in type I interferon pathway, human mediator of IRF3 activation (MITA) but not the murine homologue MPYS, was cleaved in cells infected with DEN-1 or DEN-2 and with expression of the enzymatically active protease NS2B3. The cleavage site of MITA was mapped to LRR↓^96^G and the function of MITA was suppressed by dengue protease. DEN replication was reduced with overexpression of MPYS but not with MITA, while DEN replication was enhanced by MPYS knockdown, indicating an antiviral role of MITA/MPYS against DEN infection. The involvement of MITA in DEN-triggered innate immune response was evidenced by reduction of IRF3 activation and IFN induction in cells with MITA knockdown upon DEN-2 infection. NS2B3 physically interacted with MITA, and the interaction and cleavage of MITA could be further enhanced by poly(dA:dT) stimulation. Thus, we identified MITA as a novel host target of DEN protease and provide the molecular mechanism of how DEN subverts the host innate immunity.

## Author Summary

The pathogenesis of severe dengue diseases remains unclear, but magnitude of dengue virus (DEN) replication is believed to be one of the major determining factors. Thus, revealing how DEN evades the host defense mechanism such as type I interferon (IFN) system is important for better understanding this devastating disease. Although several DEN viral proteins have been reported as IFN-resistant factors, without knowing the cellular targets, the mechanism of how DEN subverts IFN system is poorly understood. In this study, we found that the human mediator of IRF3 activation (MITA), also known as STING and ERIS, was cleaved in cells infected with DEN and in cells expressing an enzymatically active DEN protease NS2B3. MITA is known as a DNA sensor for IFN production and its antiviral role has also been demonstrated for several DNA and RNA viruses. DEN protease appears to cleave MITA but not its murine homologue MPYS, and this cleavage resulted in impaired MITA activation. Ectopic overexpression of MPYS but not MITA reduced DEN replication, and knockdown of endogenous MPYS enhanced DEN replication. Thus, we find that MITA/MPYS is involved in host defense against DEN replication and DEN protease targets MITA to subvert its antiviral effect.

## Introduction

Dengue has emerged as a rapidly spreading vector-borne disease annually affecting 50 to 100 million people living in tropical and subtropical areas [1], [2]. Dengue virus (DEN) infection of humans causes a spectrum of illnesses ranging from mild classical dengue fever to severe dengue hemorrhagic fever (DHF) and dengue shock syndrome (DSS). The pathogenesis of severe dengue diseases remains unclear, but magnitude of DEN replication is believed to be one of the major determining factors [3]. Type I interferons (IFNs), mainly IFNα and IFNβ, play central roles in host defense against viral infection [4], [5]. DEN replication was sensitive to IFN in both cell-based assays and infected animals [6], [7], and the IFN-induced 2′,5′-oligoadenylate synthetase (OAS)/RNase L pathway may contribute to host defense against DEN infection [8]–[10]. Thus, for DEN to survive and replicate in host cells, it likely needed to evolve a way to downregulate the cellular IFN system.

DEN triggers IFNβ through a molecular mechanism involving the retinoic acid inducible gene I (RIG-I) signaling pathway [11], [12]. RIG-I binding to viral RNA triggers conformational changes that expose the N-terminal caspase recruitment domain (CARD) [13]. Mitochondrial antiviral signaling (MAVS) [14], [15], also called VISA [16], IPS-1 [17], and Cardif [18], relays the signal to activate the downstream kinases, thus resulting in activation of IFN regulatory factor 3 (IRF3), IRF7, and NF-κB, and finally IFN production [13], [19]. DEN is known to be a weak IFN inducer [11], [20] and MAVS is cleaved by caspases in DEN-infected cells [21]. Furthermore, IFN induction in response to poly(I:C) transfection and infection by several viruses such as Newcastle disease virus, Sendai virus (SeV), and Semliki Forest virus was reduced in DEN-infected human dendritic cells [22]. A catalytically active DEN NS2B3 protease was found to reduce the IFNβ promoter activation triggered by SeV infection and poly(I:C) transfection [22]. However, the molecular target of dengue protease in IFN induction remains elusive.

Mediator of IRF3 activation (MITA) [23], also known as stimulator of interferon genes (STING) [24], endoplasmic reticulum IFN stimulator (ERIS) [25], and transmembrane protein 173 (TMEM173), shares 81% similarity (68% identity) with its murine homologue MPYS [26]. MITA is a membrane protein involved in IFN production triggered by viral RNA and dsDNA [23]–[25]. MITA interacts with RIG-I, forms a complex with MAVS, activates IRF3 phosphorylation, and is required for IFN induction triggered by RNA and DNA viruses [23], [24], [27]. MITA is positively and negatively regulated by multiple mechanisms. Phosphorylation of MITA by TBK1 is critical for virus-induced IRF3 activation [23]. K63-linked ubiquitination of MITA by TRIM56 induces MITA dimerization, and then recruits TBK1 for subsequent IFN induction [28]. MITA is downregulated by RNF5, an E3 ubiquitin ligase, which targets MITA for ubiquitination and degradation [29]. MITA association with TBK1 with dsDNA stimulation was downregulated by Atg9a, an autophagy related protein [30]. Interestingly, amino acids 125–222 of MITA exhibit homology to flaviviral NS4B proteins, and yellow fever virus NS4B has been shown to suppress MITA-mediated IFN production [27].

To understand the molecular target of dengue protease in IFN pathway, we investigated the cleavage of MITA and MPYS in cells infected with DEN or with expression of DEN protease. MITA, but not murine homologue MPYS, was cleaved in a DEN protease-dependent manner, which led to impaired IRF3 activation stimulated by Japanese encephalitis virus (JEV) infection or by transfection with poly(I:C) or poly(dA:dT). The potential cleavage site on MITA was mapped and the possible regulatory mechanisms and biological significance of torpid MITA signaling by dengue protease are discussed.

## Results

### Dengue NS2B3 suppresses virus-, dsRNA-, and dsDNA-induced IRF3 activation

**Figure 1:**
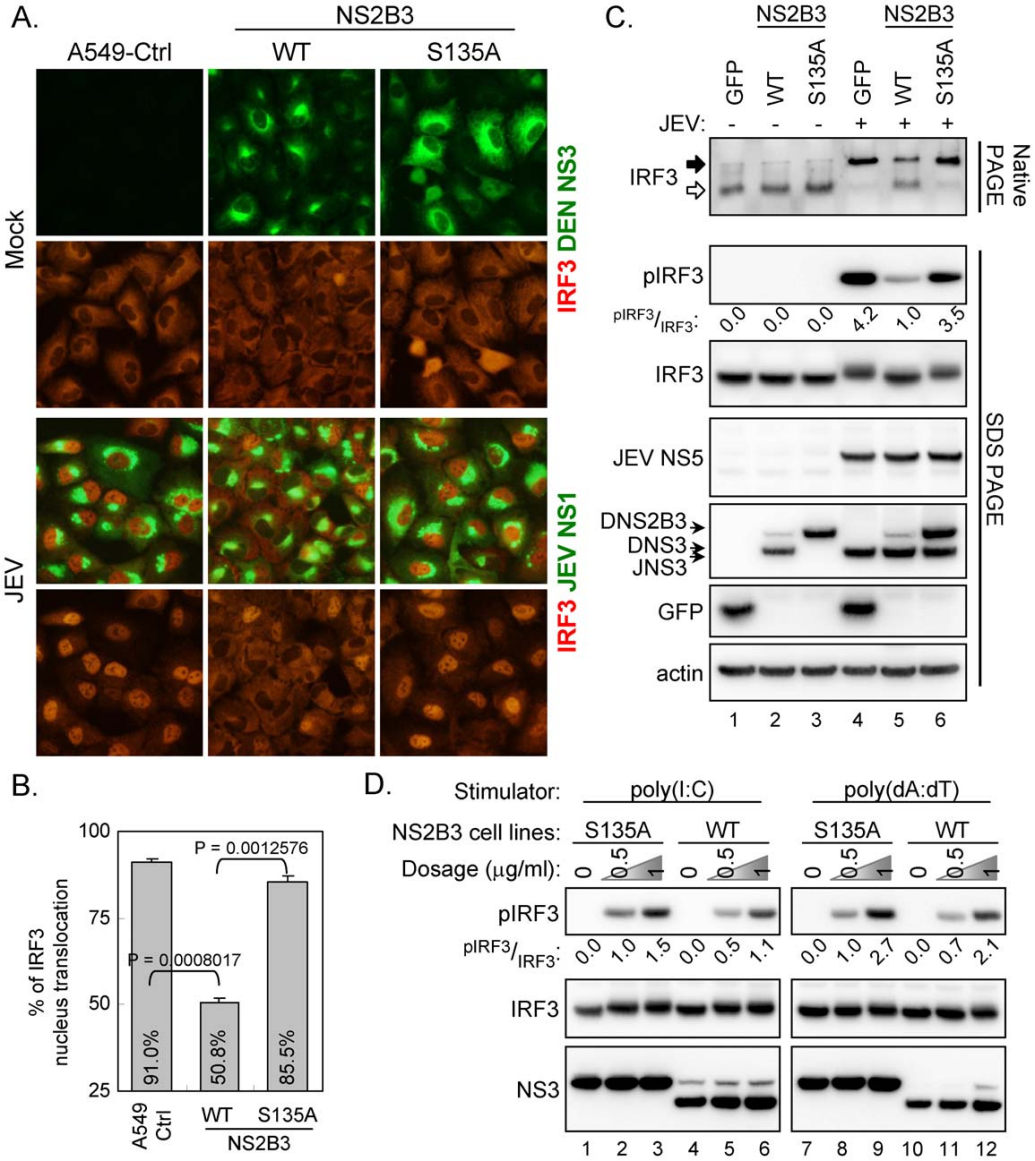
Suppression of IRF3 activation by dengue NS2B3 depends on its protease activity. (A) A549 cells were stably transduced with lentivirus expressing dengue NS2B3 (WT) or its protease-dead mutant S135A. Cells were mock infected or infected with JEV (multiplicity of infection [MOI] 5) for 24 h, and then fixed for immunofluorescence assay (IFA). Green, viral proteins as indicated; red, endogenous IRF3. (B) The percentage of IRF3 nucleus translocation were determined from three independent experiments and shown as the mean and SD. Data of the indicated groups were compared by two-tailed Student's *t* test. (C) IRF3 activation was analyzed by IRF3 dimerization with native PAGE (black arrow, dimer; open arrow, monomer) and immunoblotting with antibodies against S396 phospho-IRF3 (pIRF3) and IRF3 by SDS-PAGE as indicated. Western blotting was also performed with antibodies against JEV NS5, DEN/JEV NS3, GFP, and actin. (D) Western blot analysis of IRF3 and pIRF3 in stable cell lines expressing the wild-type or S135A-mutated dengue NS2B3 transfected with poly(dA:dT) or poly(I:C) at the indicated dose. The band density was quantified with ImageJ and the relative ratios of pIRF3 to IRF3 are shown in panels C and D. The western blot results are the representative data from three independent experiments.

DEN antagonizes type I IFN response in human dendritic cells through a catalytically active protease complex, NS2B3 [22]. To further address the role of dengue protease in suppressing the IFN pathway, we established a stable DEN-2 protease NS2B3-overexpressing A549 cell line by lentiviral transduction. A single point mutation changing serine residue 135 of NS3 to alanine (S135A) abolished protease activity, as evidenced by loss of NS2B3 autocleavage (Figure 1C and 1D). JEV, known to induce high level of IRF3 activation and trigger IFN induction [11], whose replication was not affected by dengue protease (Figure S2) was used as a viral IFN inducer in this study. In contrast to control cells overexpressing GFP, cells overexpressing dengue NS2B3 showed reduced phosphorylation of IRF3 triggered by JEV infection (Figure S1). JEV-induced IRF3 nucleus translocation (Figure 1A, 1B and Table S1), dimerization (Figure 1C), and phosphorylation (Figure 1C), were all reduced in cells overexpressing the wild-type but not enzyme-dead (S135A) NS2B3, under similar levels of JEV replication (Figure S2).

To ascertain the spectrum of this inhibitory effect of dengue protease, we transfected cells with poly(dA:dT) and poly(I:C), two synthetic analogs of dsDNA and dsRNA, respectively, that mimic foreign nucleic acids from pathogens. Cells overexpressing the enzymatically active dengue NS2B3 showed attenuated IRF3 phosphorylation triggered by poly(dA:dT) or poly(I:C) transfection (Figure 1D). Therefore, virus-, DNA- and RNA-triggered IRF3 activation could be subverted by dengue NS2B3 in a protease activity-dependent manner.

### MITA is the target of dengue protease NS2B3

**Figure 2:**
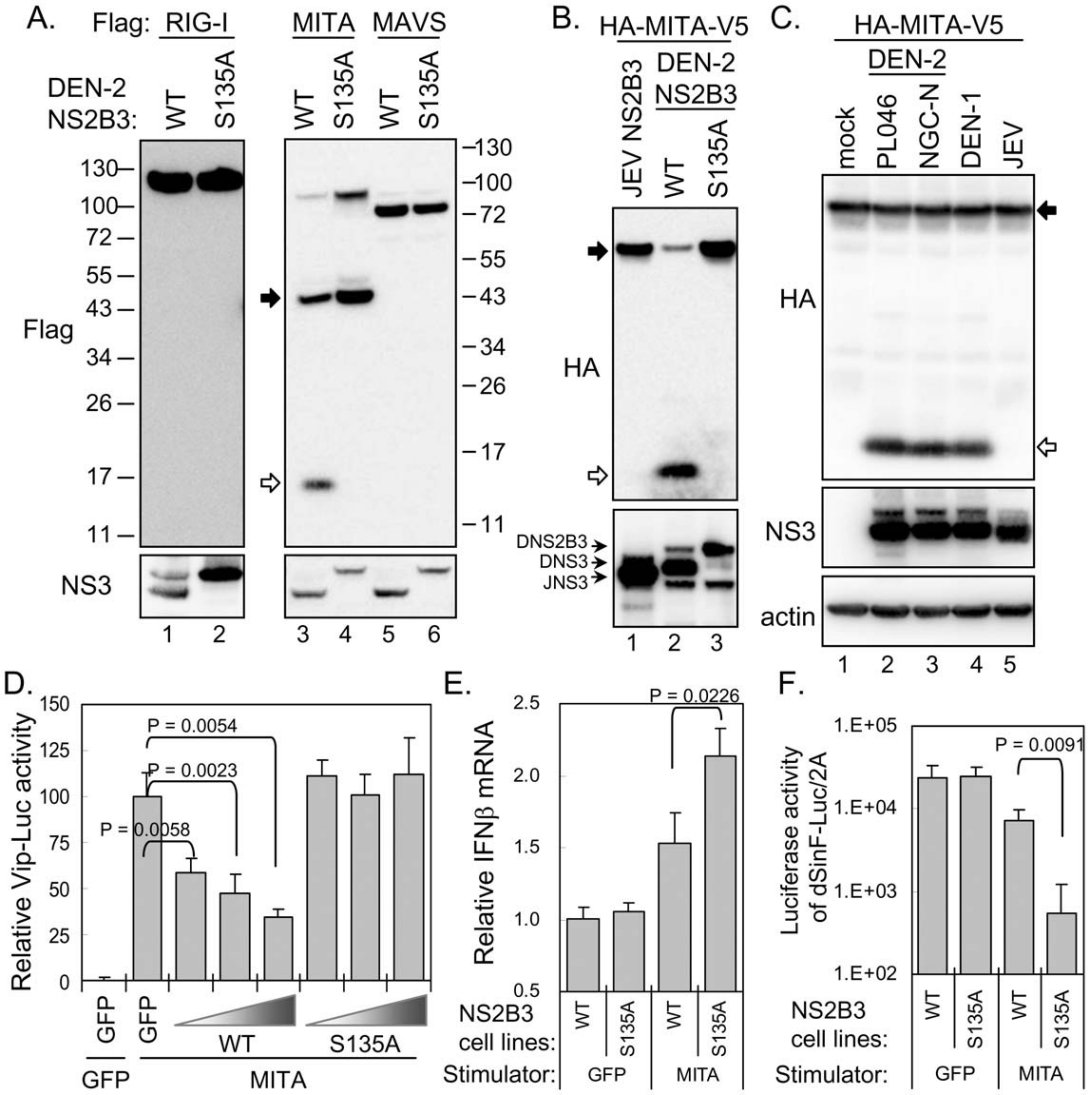
MITA is targeted by dengue protease. (A) N-terminal Flag-tagged RIG-I, MITA, or MAVS was cotransfected with dengue NS2B3 (DNS2B3) in 293T/17 (lanes 1 and 2) or A549 (lanes 3–6) cells. Cells were harvested for western blotting at 18 h post transfection with the indicated antibodies. Molecule weight (kDa) markers are shown on the sides. The position for full-length MITA is indicated by a black arrow and for the cleaved MITA by an open arrow. (B) A549 cells were cotransfected with HA-MITA-V5 plus the viral protease encoded by JEV or DEN-2 (WT and S135A) and analyzed by immunoblotting with antibodies against HA-tag and NS3. (C) A549 cells stably expressing HA-MITA-V5 were infected with the indicated virus (MOI 5) for 24 h and then analyzed by immunoblotting with antibodies against HA-tag, NS3, and actin. (D) A549 cells were cotransfected with HA-MITA-V5 (0.3 µg), Vip-Luc (0.15 µg), IRF3/pCR3.1 (0.15 µg), pRL-TK (0.05 µg), and different doses of WT or S135A-mutated dengue NS2B3 (0.3, 0.45, or 0.6 µg) for 24 h. GFP plasmid was used as transfection plasmid control. The cell lysates were harvested and analyzed by dual-luciferase assay. *Firefly* luciferase activity was normalized to that of *Renilla* luciferase. The relative luciferase activity to that of cotransfection of GFP plus MITA was calculated. Data are expressed as mean and SD (*n = 3* per group), and were compared by two-tailed Student's *t* test. (E) Quantification of endogenous IFNβ mRNA levels by RT-qPCR. The WT and S135A NS2B3-expressing A549 cells were transfected with MITA or GFP for 24 h and harvested for RT-qPCR of IFNβ and actin. Data are expressed as mean and SD (*n = 3* per group), and were compared by two-tailed Student's *t* test. (F) Vero cells were pretreated with culture medium derived from NS2B3-expressing A549 cells transfected with MITA or GFP as indicated. The conditioned Vero cells were infected with dSinF-Luc/2A (500 pfu/well) for 24 h and then harvested for luciferase assay. Data are expressed as mean and SD (*n = 3* per group), and were compared by two-tailed Student's *t* test.

To reveal the molecular target of dengue protease in the IFN pathway, three key molecules in pattern recognition receptor (PRR) signaling, RIG-I, MAVS, and MITA, were cotransfected with the wild-type (WT) or S135A-mutated NS2B3. The protein expression of RIG-I and MAVS appeared to be similar in cells with WT or S135A-mutated NS2B3 overexpression (Figure 2A). However, the protein bands corresponding to the full-length and the dimer [25] form of MITA were reduced in cells cotransfected with the wild-type but not S135A-mutated NS2B3, and an extra protein band of smaller size was detected in NS2B3(WT)-transfected cells (Figure 2A), indicating a cleavage of MITA in dengue protease-overexpressing cells. This MITA-cleavage event seems to be specific for dengue protease, because JEV protease, which shares similar substrate sequences as DEN, did not cleave MITA (Figure 2B). The cleavage of MITA could also be detected in cells infected with DEN-2, PL046 and NGC-N strains or with DEN-1 Hawaii strain but not with JEV (Figure 2C).

Importantly, this cleavage impaired the normal function of MITA, because the *viperin* promoter-driven reporter, Vip-Luc, with expression under the control of ISRE [31], was dose-dependently downregulated by cotransfection with the wild-type but not S135A-mutated dengue NS2B3 (Figure 2D). The MITA-triggered activation of the IFNβ promoter-driven reporter p125-Luc (Figure S3) and the induction of endogenous IFNβ mRNA (Figure 2E) were also reduced by dengue protease. However, Vip-Luc and p125-Luc triggered by TBK1, a downstream kinase of IFN pathway [23], [24], were not affected by dengue protease (Figure S4).

Furthermore, culture medium derived from A549 cells transfected with MITA plus S135A-mutated NS2B3 expression showed a stronger anti-VSV status than that from MITA plus the wild-type NS2B3 in Vero cells (Figure S5). We further used an IFN-sensitive recombinant sindbis virus containing a *Firefly luciferase* reporter gene (dSinF-Luc/2A) [32] to quantify the impact of DEN antagonizing antiviral activity. As expected, the luciferase activities derived from dSinF-Luc/2A were dose-dependently reduced by IFNα treatment (Figure S6A). Furthermore, dSinF-Luc/2A replicated to higher level in cells pre-infected with DEN-2 (Figure S6B), supporting the notion that DEN-2 dampens IFN pathway. Consistent with the VSV results (Figure S5), culture medium derived from cells with transfection of MITA and S135A-mutated NS2B3 expression showed a stronger antiviral activity against dSinF-Luc/2A as compared to that from transfection of MITA with wild-type NS2B3 expression (Figure 2F). Our results suggest that DEN infection subverts the innate IFN immunity by cleaving MITA through a dengue protease-dependent mechanism.

### The residues 93–96 (LRRG) of MITA are important for dengue protease cleavage

**Figure 3:**
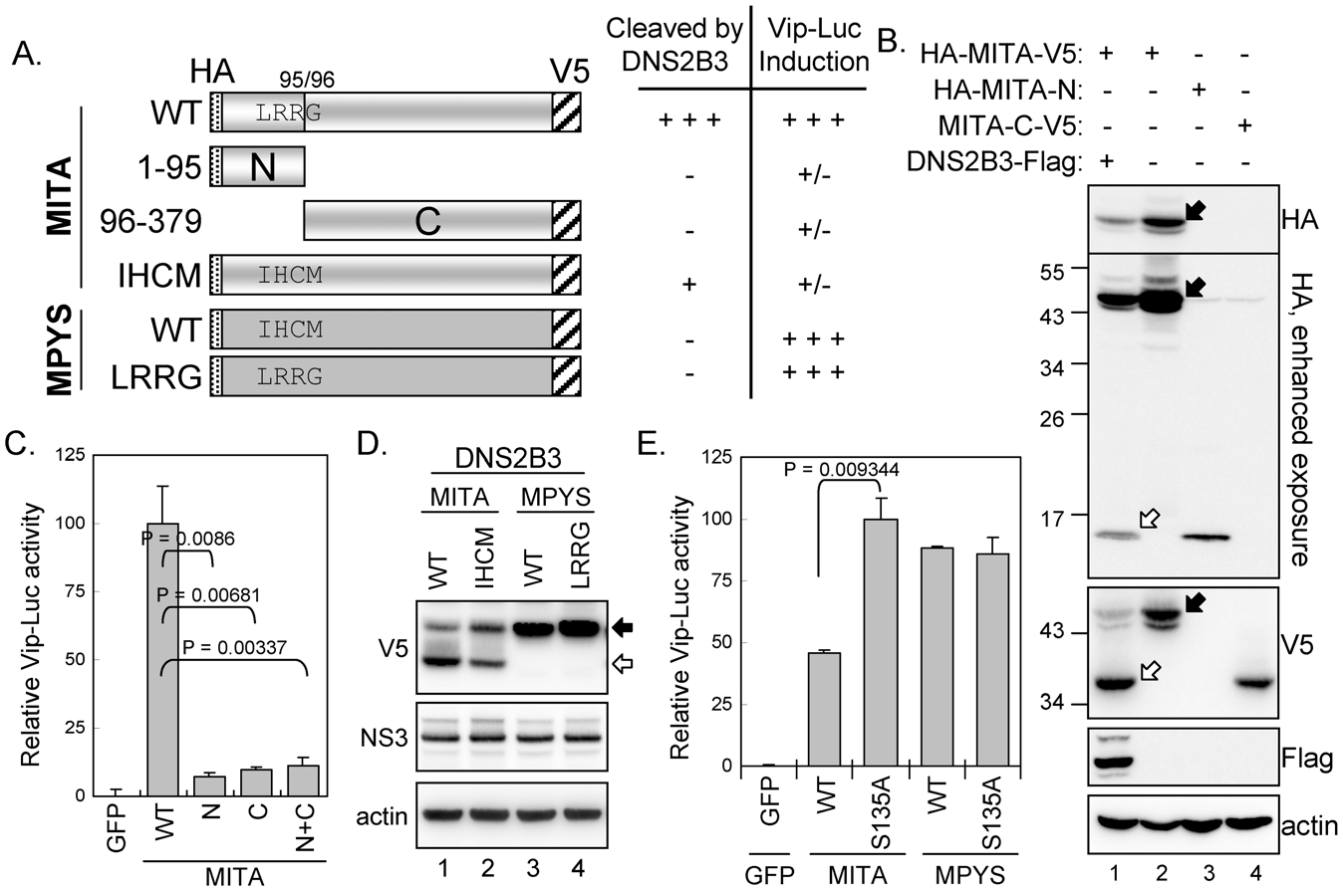
Mapping of the dengue protease cleavage site of MITA. (A) Schematic diagram and summarized properties of MITA constructs. Constructs were N-terminal HA- and C-terminal V5-tagged and are numbered according to the amino acid residues. The potential cleavage site LRRG in human MITA and the corresponding sequence IHCM in murine MPYS are indicated. (B) A549 cells were transfected with the full-length or deletion constructs of MITA with or without the Flag-tagged dengue NS2B3. Transfectants were harvested for immunoblotting with antibodies indicated at the right. The positions for full-length MITA are indicated by black arrows and the cleaved MITA by open arrows. (C) A549 cells were cotransfected with Vip-Luc (0.2 µg), IRF3/pCR3.1 (0.3 µg), pRL-TK (0.1 µg), plus GFP control or the indicated MITA constructs (0.4 µg) for 24 h. The cells were harvested and analyzed by dual-luciferase assay. The relative normalized luciferase activities are expressed as mean and SD (*n = 3* per group), and were compared by two-tailed Student's *t* test. (D) Immunoblotting of A549 cells cotransfected with DEN-2 NS2B3 plus the indicated constructs of MITA or MPYS for 24 h. (E) Dual-luciferase assay of A549 cells cotransfected with Vip-Luc (0.15 µg), IRF3/pCR3.1 (0.15 µg), pRL-TK (0.05 µg), plus the wild-type or S135A-mutated dengue NS2B3 (0.35 µg) with MITA or MPYS (0.3 µg) for 24 h. GFP was used as the negative control. The cells were harvested and analyzed by dual-luciferase assay. Data are expressed as mean and SD (*n = 3* per group), and were compared by two-tailed Student's *t* test.

To determine the potential cleavage site of dengue protease in MITA, we added an HA-tag and a V5-tag to the N and C termini, respectively, of MITA (Figure 3A). Western blot analysis with antibodies against the tags showed that the N- and C-terminal fragments of MITA produced with NS2B3 cotransfection were ∼1/4 (∼12 kDa) and ∼3/4 (∼35 kDa) of the full-length MITA (379 amino acids, ∼47 kDa with tags), respectively (Figure 3B). Because the consensus cleavage sites for dengue proteases are two basic residues followed by a small amino acid, we suspected LRR↓^96^G as the most likely cleavage site of MITA by dengue NS2B3. We constructed plasmids expressing the expected cleavage products: HA-MITA-N for the N-terminal residues 1–95 plus an HA tag and MITA-C-V5 for the C-terminal residues 96–379 plus a V5 tag. The smaller fragments of MITA produced by NS2B3 cotransfection co-migrated with HA-MITA-N (a.a. 1–95) and MITA-C-V5 (a.a. 96–379) (Figure 3B), suggesting that LRR↓^96^G, located between the second and the third transmembrane domain of MITA, is likely the site cleaved by dengue NS2B3. To understand the influence of this cleavage on MITA function, we determined whether these truncated MITAs were still able to transactivate the *viperin* promoter. Cotransfection of Vip-Luc with the MITA deletion constructs revealed that neither MITA-N nor MITA-C could trigger Vip-Luc expression as did the full-length MITA (Figure 3C), so cleavage of MITA by dengue protease would dampen its normal cellular function.

We further checked whether the murine homolog of MITA, MPYS (Gene ID:72512) [23]–[26], is sensitive to dengue protease. Different from the results for MITA, the expression pattern of MPYS was not changed by cotransfection with dengue NS2B3 (Figure 3D). Furthermore, replacing the LRRG sequences of MITA with the corresponding sequence IHCM found in MPYS reduced the cleavage of MITA by dengue NS2B3 (Figure 3D), which supports LRR↓^96^G as the target site of dengue protease in MITA. However, changing only one residue of the LRRG motif, L93I, R94A, R95A and G96P mutation, did not confer resistance to dengue protease of MITA (Figure S7). We also noted that some of these MITA mutants such as IHCM, R94A, R95A, and G96P greatly lost their ability to trigger Vip-Luc expression, suggesting that this LRRG region is important for MITA's function (Figure S7). To test whether introducing the cleavage site LRRG from MITA to MPYS would make MPYS susceptible to DEN protease, we made a MPYS-LRRG construct by replacing the IHCM sequences of MPYS with LRRG. MPYS-LRRG was still resistant to the cleavage of DEN protease (Figure 3D).

To address the interplay of dengue protease with MITA versus MPYS, we used immunoprecipitation-immunoblotting (IP-western) assay to determine whether these proteins physically interact with each other. Cells were cotransfected with V5-tagged MITA or MPYS plus the Flag-tagged enzyme-dead (S135A) dengue protease. Immunoprecipitation of MITA with anti-V5 antibody co-precipitated NS2B3 (Figure S8). DEN protease interacted with MITA, and to a lesser extent also with MITA-IHCM-mutant, but not much with WT and LRRG-mutant of MPYS (Figure S8). Cotransfection of MITA with dengue NS2B3 suppressed more than 50% of Vip-Luc activity as compared to transfection with the enzyme-dead S135A mutant; while Vip-Luc triggered by the dengue protease-resistant MPYS remained unaffected by NS2B3 cotransfection (Figure 3E). Therefore, dengue protease subverts innate immunity by cleavage of human MITA.

### MPYS but not MITA downregulates DEN replication

**Figure 4:**
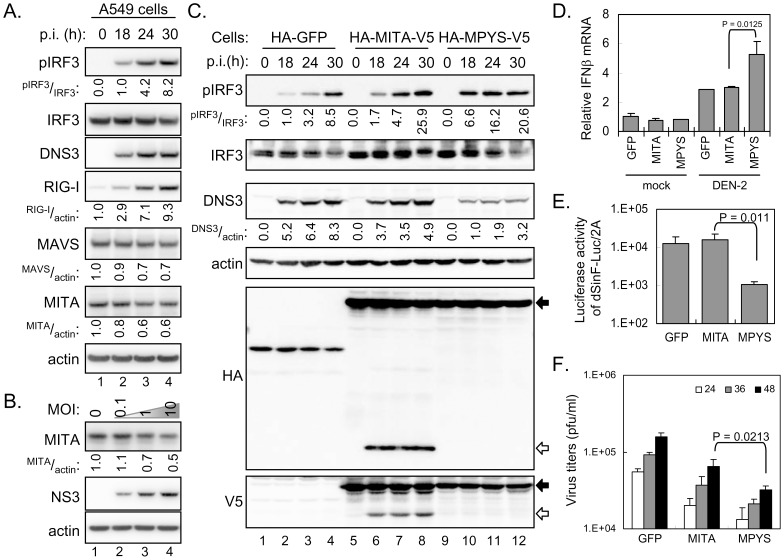
DEN replication is reduced by MPYS but not MITA. (A) A549 cells infected with DEN-2 (MOI 5) for various times were harvested for western blot analysis. Immunoblotting was done with antibodies against pIRF3, IRF3, DEN NS3, RIG-I, MAVS, MITA, and actin as indicated. The band density was quantified with ImageJ and the relative ratios of the indicated proteins are shown. (B) A549 cells infected with DEN-2 for 30 h with the indicated MOI were harvested for western blot analysis. (C) Immunoblotting of A549 stable cell lines expressing HA-MITA-V5, HA-MPYS-V5, or HA-GFP control infected with DEN-2 (MOI 10) for various times. The relative ratios of pIRF3/IRF3 and DEN-2 NS3/actin were analyzed as described in panel A. The positions of full-length MITA and the cleaved MITA are indicated by black arrows and open arrows, respectively. (D) IFNβ mRNA expression levels in A549 cells with GFP, MITA or MPYS overexpression were quantified by RT-qPCR after DEN-2 infection for 24 h. (E) The conditioned medium collected from DEN-2-infected cell lines expressing GFP, MITA or MPYS was analyzed for antiviral activity against IFN-sensitive dSinF-Luc/2A as described in Materials and Methods. (F) DEN-2 virus production from A549 cells with GFP, MITA or MPYS overexpression was determined by plaque forming assays at 24, 36, and 48 h post infection. The data in panels D, E, and F are mean and SD (*n = 3* per group), and were compared by two-tailed Student's *t* test.

To determine whether the endogenous MITA is targeted by DEN infection, we detected the protein levels of MITA and several IFN signaling molecules in DEN-infected cells by western blotting (Figure 4A). As expected, IRF3 phosphorylation and expression of DEN viral protein NS3 and IFN-induced RIG-I increased with DEN-2 infection. Furthermore, not only for MAVS that is cleaved by DEN-2-induced caspases [21], the endogenous MITA protein levels were also reduced in cells with DEN-2 infection through an infection time- and infection dose-dependent manner (Figure 4A and 4B).

To ascertain whether cleavage of MITA discredits innate immunity in response to DEN infection, we established stable A549 cells overexpressing MITA or MPYS by lentiviral transduction. MITA is a DNA sensor and plasmid transfection activates its signaling [24], [27], however different from the transient transfection, cells stably expressing MITA or MPYS showed no sign of basal IRF3 activation (Figure 4C). Upon stimulation with dsDNA, we noted higher IRF3 phosphorylation triggered by poly(dA:dT) in A549 cells expressing MITA or MPYS than the GFP control (Figure S9). In response to DEN-2 infection, cells with MPYS overexpression showed higher levels of IRF3 phosphorylation and lower dengue viral NS3 protein expression than with the GFP control (Figure 4C). However, in MITA-overexpressing cells, MITA was cleaved, as indicated by the smaller protein fragments recognized by anti-HA and anti-V5 antibodies (Figure 4C) and no anti-DEN effect of MITA was noted because of similar levels of dengue viral NS3 protein expression between MITA and control GFP cells (Figure 4C). Furthermore, the cleaved MITA products, HA-MITA-N or MITA-C-V5, had no effect on IRF3 phosphorylation, no anti-DEN activity, and no further cleavage (Figure S10). Consistent with the viral protein data detected by western blotting, less infectious DEN-2 production was noted in MPYS-expressing cells that had higher IFNβ expression level (Figure 4D) and stronger antiviral activity against IFN-sensitive dSinF-Luc/2A (Figure 4E), as compared with the MITA-expressing cells (Figure 4F).

### Silencing MITA/MPYS attenuates host antiviral signaling

**Figure 5:**
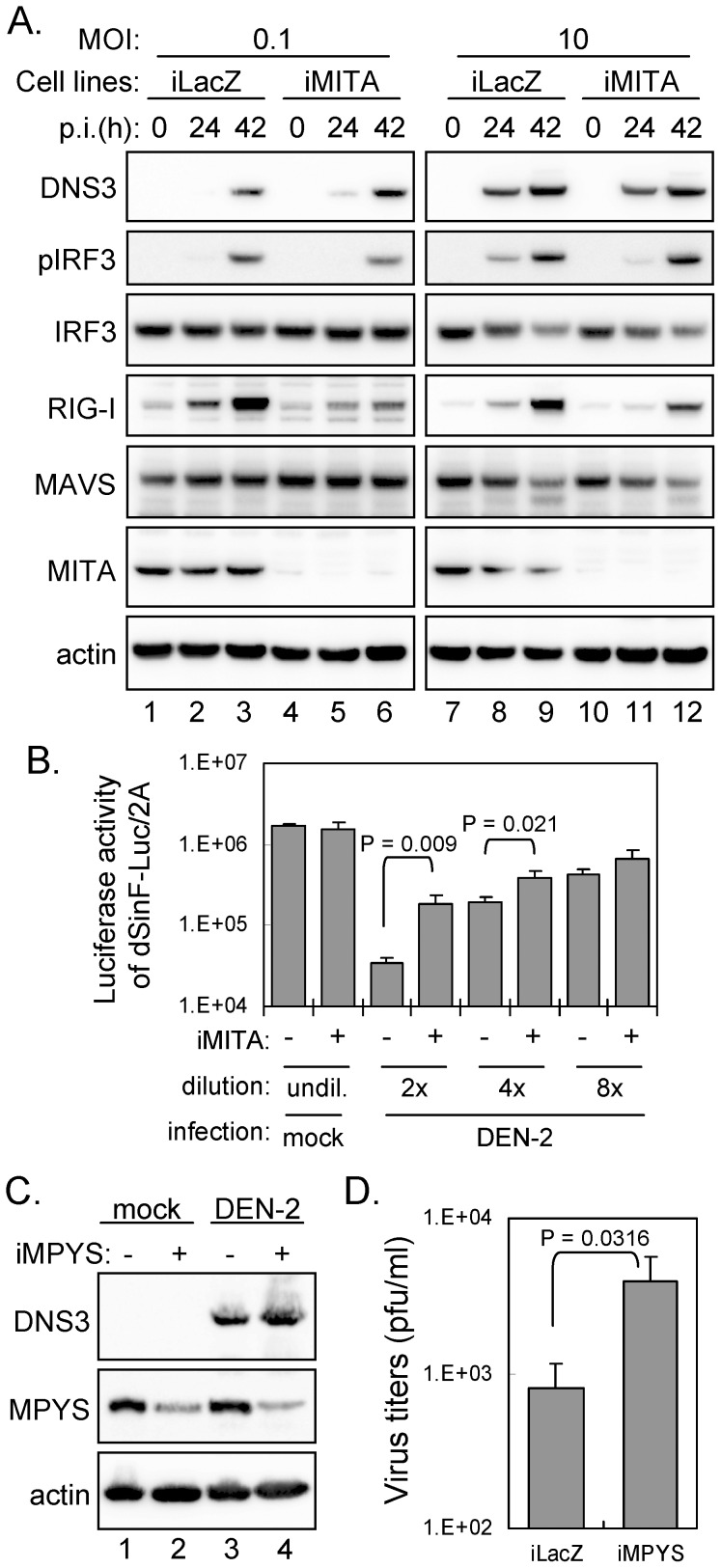
Silencing MITA/MPYS attenuates host antiviral signaling. (A) Human A549 cells stably expressing shRNA targeting control *LacZ* or *MITA* were infected with DEN-2 (MOI 0.1 and 10) for various times. Immunoblotting was performed with antibodies against DEN NS3, pIRF3, IRF3, RIG-I, MAVS, MITA, and actin. (B) The conditioned medium collected from mock or DEN-2-infected (MOI 10, 24 h p.i.) iLacZ or iMITA cells was analyzed for antiviral activity against IFN-sensitive dSinF-Luc/2A as described in Materials and Methods. Data are expressed as mean and SD (*n = 3* per group), and were compared by two-tailed Student's *t* test. (C and D) Murine Hepa 1–6 cells stably expressing shRNA targeting control *LacZ* or *MPYS* were infected with DEN-2 (MOI 5) for 24 h. Cell lysates were analyzed by western blotting with indicated antibodies (C) and culture supernatants were harvested for DEN-2 virus titration by plaque forming assays (*n* = 3) (D).

To further address the role of endogenous MITA/MPYS in DEN infection, we knocked down the endogenous MITA expression in A549 cells by lentivirus-delivered shRNA targeting human MITA gene. A slight increase of DEN replication was noted in iMITA cells, especially when a low MOI was used (Figure 5A and Figure S11), probably reflecting that high MOI of DEN infection blunts MITA efficiently and MITA knockdown has little additional effect. We also noted that the levels of IRF3 phosphorylation and IFN-induced RIG-I expression were reduced in iMITA cells upon DEN-2 infection (Figure 5A), supporting the notion that MITA plays a role in IFN signaling during DEN infection. Consistent with the protein expression data (Figure 5A), culture medium derived from DEN-2-infected iMITA cells also exhibited lower antiviral activity against dSinF-Luc/2A (Figure 5B) as compared to control knockdown cells. To further demonstrate the contribution of endogenous MPYS on anti-DEN host defense, we reduced the endogenous MPYS expression of murine Hepa 1–6 cells that has been used for DEN study [33] by shRNA targeting MPYS. DEN-2 viral protein expression (Figure 5C) and viral progeny production (Figure 5D) were enhanced in cells with reduced MPYS, further supporting the antiviral role of MITA/MPYS and the need of DEN to subvert it by protease degradation.

### Cellular distribution of MITA/MPYS upon stimulation

**Figure 6:**
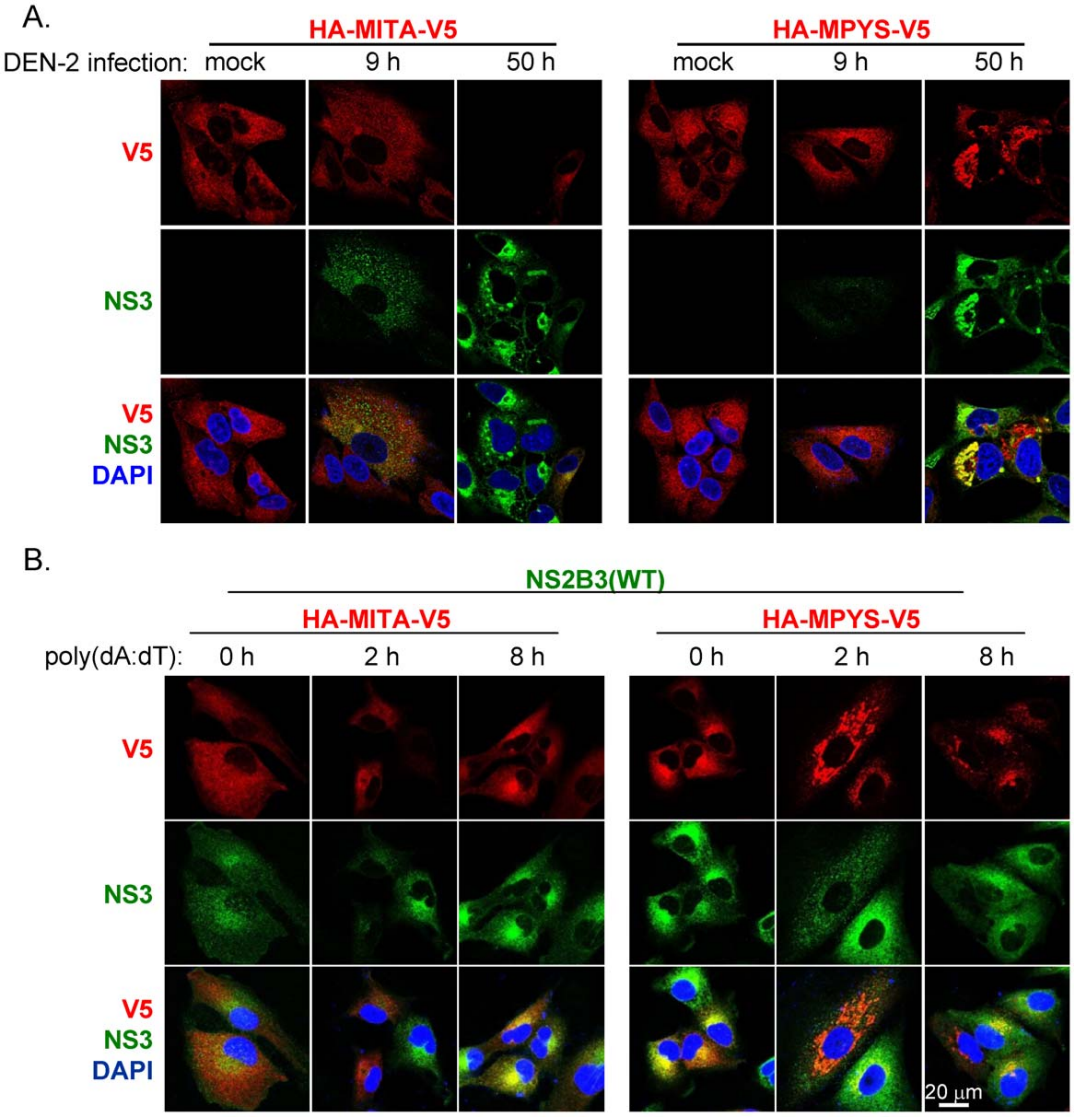
Cellular distribution patterns of MITA and MPYS upon stimulation. (A) A549 stable cell lines expressing HA-MITA-V5 or HA-MPYS-V5 were infected with DEN-2 (MOI 20) for the indicated times and then fixed for IFA. Red, overexpressed MITA or MPYS stained with anti-V5 antibody; green, DEN-2 NS3; blue, DAPI staining. IFA were analyzed by confocal laser scanning microscopy. (B) A549 stable cell lines overexpressing dengue NS2B3(WT) plus MITA or MPYS were transfected with poly(dA:dT) (0.5 µg/ml) for 0, 2, and 8 h and analyzed by IFA as described in panel A.

MITA is known to be critical for intracellular DNA-mediated IFN production but its role in dsRNA-triggered IFN production is more controversial [27]. Since DEN is a RNA virus, we are interested to know whether MITA/MPYS is activated in DEN-infected cells. Because MITA forms cytoplasmic punctate structures during activation [30], we determined the cellular distribution of MITA and MYPS in DEN-infected cells. To avoid using dsDNA plasmid transient transfection that activates MITA, we used A549 cells stably expressing MITA or MPYS to examine the cellular localization of MITA/MPYS. At the early time point of DEN-2 infection, both MITA and MPYS showed homogenous cytoplasmic distribution. However, at the later time point, MITA was diminished likely through cleavage by DEN protease and then degradation by cell machinery, while MPYS formed punctate structures, suggesting that MPYS is activated by DEN-2 infection (Figure 6A). We then established stable cells overexpressing the wild-type NS2B3 plus MITA or MPYS by lentivirus transduction. In the absence of stimulation, MITA/MPYS and dengue protease co-existed in the same cells (Figure 6B) even though MITA is cleavable by NS2B3, suggesting that certain stimulation is required to facilitate this cleavage event. With dsDNA stimulation, MPYS formed punctate structures but not MITA with NS2B3 expression (Figure 6B), supporting the notion that MITA but not MPYS is targeted by dengue protease.

### MITA interacts with dengue protease, and dsDNA further enhances this interaction

**Figure 7:**
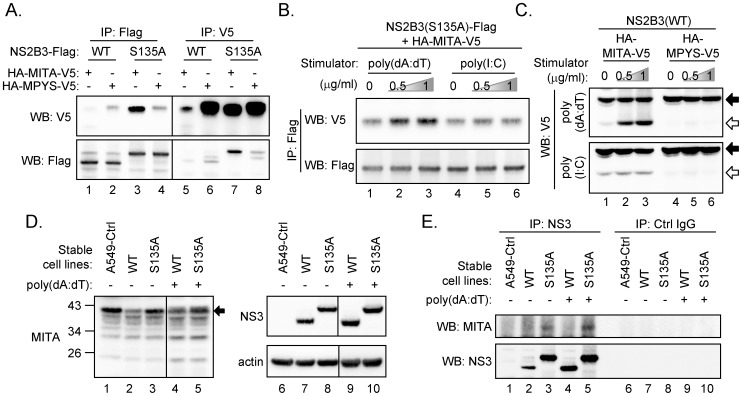
Dengue protease NS2B3 interacts with MITA. (A) Immunoprecipitation and western blot analysis (IP-WB) of 293T/17 cells cotransfected with dengue NS2B3 (WT or S135A-mutated) plus MITA or MPYS for 18 h with the indicated antibodies. (B) IP-WB analysis of 293T/17 cells cotransfected with S135A-mutated NS2B3 and MITA for 48 h, then the cells were treated with different doses of poly(dA:dT) or poly(I:C) as indicated for 18 h. (C) Western blot analysis of A549 stable cell lines overexpressing dengue NS2B3(WT) plus MITA or MPYS treated with poly(dA:dT) or poly(I:C) (0, 0.5, and 1 µg/ml) for 24 h with antibody against V5-tag. Black arrow, full-length MITA; open arrow, cleaved MITA. A549 stable cell lines overexpressing dengue NS2B3 (WT or S135A) were stimulated with poly(dA:dT) (0.5 µg/ml) for 24 h and then analyzed by western blotting (D) and by IP-WB (E) analysis with the indicated antibodies. Black arrow indicates the full-length endogenous MITA.

To further address the interplay of dengue protease with MITA versus MPYS, cells were cotransfected with V5-tagged MITA or MPYS plus the Flag-tagged WT or enzyme-dead (S135A) dengue protease. Immunoprecipitation of NS2B3(S135A) with anti-Flag antibody readily brought down MITA but not much of MPYS (Figure 7A). Similar results were noted by immunoprecipitation of MITA with anti-V5 antibody and then immunoblotting for NS2B3 with anti-Flag antibody (Figure 7A). Interestingly, the interaction between MITA and dengue NS2B3 was enhanced by transfection with poly(dA:dT) but not much with poly(I:C) (Figure 7B). To determine whether this interaction contributes to the cleavage event, stable cells overexpressing the wild-type NS2B3 plus MITA or MPYS were treated with poly(dA:dT) or poly(I:C). A basal level of cleavage of MITA but not MPYS was detected (Figure 7C, lanes 1 and 4), and this cleavage was further enhanced by transfection with poly(dA:dT) but not with poly(I:C) (Figure 7C).

A reduced level of the full-length endogenous MITA was noted in cells with WT but not with enzyme-dead NS2B3 (S135A) regardless of dsDNA stimulation (Figure 7D). We were not able to detect the cleaved products of MITA protein probably due to rapid degradation. The protein-protein interaction of endogenous MITA and dengue protease could be demonstrated by immunoprecipitation with anti-NS3 antibody and then western blotting with anti-MITA antibody, especially in cells with the enzyme-dead S135A NS2B3 (Figure 7E). Overall, we found that dengue protease NS2B3 targets MITA, an important signaling molecule of host innate immunity in response to foreign nucleic acids, to downregulate the host defense mechanism.

## Discussion

In this study, we found that MITA, a key adaptor molecule in host innate immune response, is targeted by the DEN protease NS2B3. MITA, also known as STING, MPYS and ERIS, is an ER-localized transmembrane protein essential for IFN induction triggered by DNA pathogens [27], [34], [35] and probably also by some RNA viruses [23], [24], [27], [36]. Targeting MITA during DEN infection may result in reduced host defense against DEN, and maybe also against DNA pathogens such as bacterial infection. Even though concurrent bacteraemia in patients with dengue fever is rare, some reports have implicated bacterial infection in severe forms of dengue diseases. For example, secondary bacteraemia was a contributor to death in 4 of the 9 adult patients who died of dengue-related illness in Singapore [37]. A study in Taiwan indicated that 5.5% of the patients with DHF/DSS had concurrent bacteremia [38], and a study of DHF infants in India showed 21% with bacterial co-infections [39]. Thus, our results showing that DEN may modulate the innate immunity predisposition to other infections provide a molecular explanation for the mortality of nosocomial bacteraemia in dengue patients.

The protease activity of DEN NS3 depends on the association with NS2B cofactor [40]–[42], and the viral NS2B3 protease cleaves the viral polyprotein precursor at the junctions of NS2A/NS2B, NS2B/NS3, NS3/NS4A, and NS4B/NS5 [40], [43]. These cleavage sites have the consensus sequence of two basic amino acids (KR, RR, RK, and occasionally QR) at the −2 and −1 positions, followed by a small amino acid (G, A, or S) at the +1 position [40], [42]–[44]. Previously, by using an IFNβ-Luc reporter assay, IFNβ promoter activation triggered by SeV infection was reduced by DEN NS2B3 through a protease-dependent mechanism [22]. In this study, we further demonstrated that DEN protease reduced IFN induction and IRF3 phosphorylation triggered by JEV infection and by transfection with poly(I:C) and poly(dA:dT). Furthermore, human MITA but not the murine homologue MPYS was cleaved in cells expressing an enzyme-active but not enzyme-dead NS2B3. From the sizes of the cleaved products, LRR↓^96^G, which matches the flaviviral protease consensus sequences, was predicted to be the cleavage site of MITA by DEN protease. Mutation of LRRG to the corresponding sequence, IHCM found in murine MPYS that cannot be cleaved by dengue protease, attenuated the cleavage pattern of MITA, suggesting that MITA is a substrate of dengue NS2B3.

However, we cannot exclude the possibility that MITA is cleaved by a yet-to-be identified cellular protease that depends on the activity of dengue NS2B3. The cleavage sites for JEV and DEN proteases share the same consensus sequences; but different from the data for DEN infection and DEN protease, JEV infection and JEV NS2B3 expression failed to trigger cleavage of MITA, suggesting that other factors, besides the presence of flaviviral protease, also govern this MITA cleavage event. Furthermore, this DEN protease-mediated cleavage of MITA could be enhanced by dsDNA stimulation, suggesting that some dsDNA-induced factor(s) also participate in this cleavage event. So far we could conclude that MITA, but not MPYS, is specifically cleaved by either DEN protease itself or by host protease(s) depending on the activity of DEN protease. This different response between human MITA and murine MPYS provides a potential clue to improve the animal models for DEN pathogenesis study.

**Figure 8:**
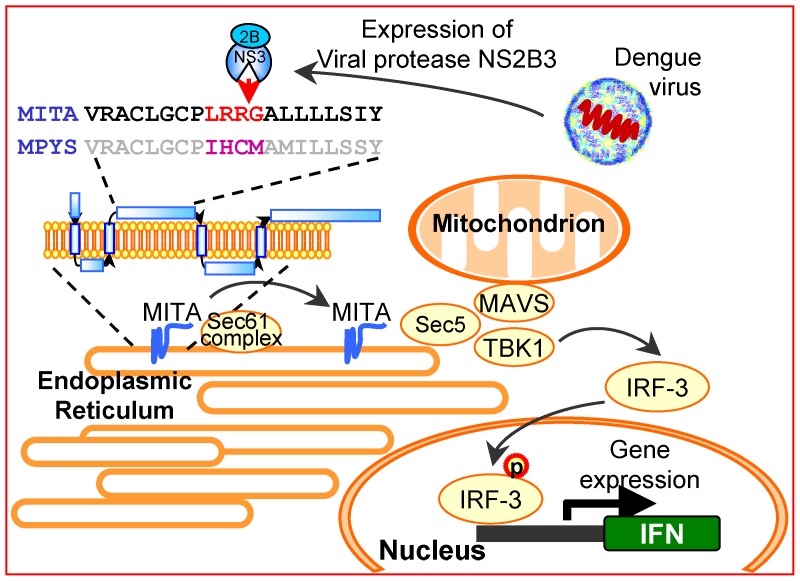
DEN antagonizes MITA-mediated antiviral signaling. Activated MITA translocates from ER to associate with Sec5 translocon complex, and then reaches the cytoplasmic punctate structures to assemble with TBK1. This activation process leads to phosphorylation and translocation of IRF3, and then induces antiviral IFN production. DEN-encoded protease NS2B3 targets human MITA at LRR↓^96^G but not the murine homologue MPYS for cleavage, thus subverts the MITA-triggered antiviral signaling.

As outlined in Figure 8, in unstimulated cells, MITA mainly localizes to the endoplasmic reticulum (ER) [24], [25], [30]. Following stimulation, MITA translocates from the ER to the Golgi and finally assembles with TBK1 in punctate membrane structures, which is required for activation of downstream signals [30]. Dengue NS3 protease also targets to ER by interacting with its cofactor NS2B [45]. Here, we found that MITA and dengue NS2B3 physically interact with each other, and this interaction could be further enhanced by stimulation with poly(dA:dT). The cleavage of MITA by NS2B3 could also be enhanced by poly(dA:dT), suggesting that NS2B3 would encounter the dsDNA-driven trafficking MITA [30] and execute the cleavage. The cleavage decreasing the function of MITA is further supported by loss of punctate structures with MITA triggered by dsDNA stimulation in cells with dengue NS2B3.

MITA is known to be critical for intracellular DNA-mediated IFN production but its role in RNA-triggered IFN production is not so clear [27]. However, MITA is required for IFN induction triggered by RNA viruses such as SeV [23] and VSV [27]. VSV [23], [24], [27] and Newcastle disease virus [25] are also sensitive to the antiviral activity mediated by MITA both *in vitro* and *in vivo*. Thus, MITA is likely not only a DNA sensor, but also involved in RNA viruses signaling. Consistent with the previous study [30], poly(I:C) did not trigger MPYS activation marker, cytoplasmic punctate structures (Figure S12), however DEN-2 infection induced MPYS punctate structures. These results suggest that poly(I:C) transfection may not completely recapitulate the events occurring during DEN infection. Several possibilities may contribute to this discrepancy, for example viral RNA may possess of property more than poly(I:C) and/or the host, both genomic and mitochondrial, DNA release in virus-infected cells would activate the MITA/MPYS signaling pathway.

DEN is a weak inducer of type I IFN, and DEN-infected human dendritic cells showed a reduced type I IFN response to infection with several viruses and to stimulation with poly(I:C) [11], [20], [22]. Several molecules of the IFN-inducing pathway are targeted by members of the *Flaviviridae* family. MAVS is cleaved by HCV NS3/4A protease [14], [18] and by caspase-1 and caspase-3 induced by DEN infection [21]. A sequence homology between flaviviral NS4B and MITA (a.a. 125–222) has been reported, and the IFN induction ability of MITA was downregulated by yellow fever virus NS4B [27]. DEN-encoded NS2B3 protease apparently also targets the human MITA. DEN replication levels were reduced in cells expressing the murine homologue of MITA, MPYS, which cannot be cleaved by dengue protease, suggesting that DEN replication benefits from MITA cleavage with dengue NS2B3. Because dengue protease is essential for DEN replication, it has been extensively investigated as an antiviral target. Whether drugs that block DEN protease can affect DEN replication and rescue the MITA-mediated innate immune response and influence DEN pathogenesis is of great interest.

## Materials and Methods

### Viruses, cells, and chemicals

DEN and JEV propagation and titration were as described previously [21], [46]. IFN-sensitive dSinF-Luc/2A sindbis virus [32] and vesicular stomatitus virus (VSV) were amplified in Vero cells as described [47]. IFNα-2a (Roferon-A) was from Roche. 293T/17 cells (ATCC, CRL-11268) and murine hepatoma Hepa 1–6 cells (ATCC CRL-1830) were cultured in DMEM medium supplemented with 10% FBS. A549, a human lung carcinoma cell line, was cultured in F-12 medium supplemented with 10% FBS. African green monkey kidney Vero cells were cultured in MEM supplemented with 10% FBS and 2 mM L-glutamine. PolyJet transfection reagent (SignaGen Laboratories) and Lipofectamine 2000 (Invitrogen) were used according to the manufacturer's instructions. Poly(dA:dT) naked and Poly(I:C) LMW were obtained from InvivoGen and delivered into cells by transfection with Lipofectamine 2000.

### Plasmids

pRK-HA-MITA and pRK-Flag-MITA were kindly provided by Dr. Hong-Bing Shu. The cDNA of *MPYS* was cloned by PCR with the primers: XhoI-mMITA(1–22): 5′-ACCGctcgagATGCCATACTCCAACCTGCATC-3′ and mMITA(1136–1114)-XbaI: 5′-CTAGtctagaCAGATGAGGTCAGTGCGGAGTGG-3′, and the sequences were identical to that of murine *MPYS* (GenBank accession no. NM_028261). To add tags to both ends of MITA, HA-MITA was subcloned into the V5-His/pcDNA3.1 vector (Invitrogen). Primers for deletions and mutations of MITA or MPYS are listed in Figure S7. The pTY lentiviral expression system was obtained from Dr. Lung-Ji Chang [48]–[50]. The lentivirus vector carrying the shRNA targeting human MITA (5′-CATGGTCATATTACATCGGAT-3′, TRCN0000160281), murine MPYS (5′-AGAGGTCACCGCTCCAAATAT-3′, TRCN0000346319) or LacZ (5′-TGTTCGCATTATCCGAACCAT-3′, TRCN0000072223), and the lentiviral vectors for cDNA overexpression, pLKO.1_AS3w.puro and pLKO.1_AS3w.bsd, were from the National RNAi Core Facility, Taiwan. Lentivirus was prepared following the protocol of the National RNAi Core Facility (Academia Sinica, Taiwan). The pCR3.1 vectors expressing DEN-2 NS2B3 and its S135A mutant have been described previously [46]. The cDNAs of NS2B3 and NS2B3(S135A) were subcloned to pTY vector for lentivirus production.

### Reporter assay

To assess the effect of DEN protease on MITA-mediated gene induction, A549 cells were cotransfected with *viperin* promoter-driven reporter Vip-Luc [31] (0.15 µg), IRF3/pCR3.1 [11] (0.15 µg), MITA/pcDNA3.1 (0.3 µg), internal control pRL-TK (Promega) (0.05 µg), and various doses of NS2B3/pCR3.1 (WT or S135A-mutant) (0.3, 0.45, and 0.6 µg) for 24 h. GFP/pCR3.1 was used as plasmid control. The cell lysates were harvested and analyzed by dual-luciferase assay system (Promega). The *firefly* luciferase activity (Vip-Luc) was normalized to that of *renilla* luciferase (pRL-TK) and the relative luciferase activities are shown.

### RT-qPCR analysis

Total RNA was prepared with an RNeasy RNA Mini Kit (Qiagen) and the cDNA was reverse transcribed from 1 µg of total RNA with random hexamer primer using a ThermoScript RT kit (Invitrogen). qPCR was then carried out using the specific primer sets for IFNβ (5′-CACGACAGCTCTTTCCATGA-3′ and 5′-AGCCAGTGCTCGATGAATCT-3′) and actin (5′-TCCTGTGGCATCCACGAAACT-3′ and 5′-GAAGCATTTGCGGTGGACGAT-3′) with the LightCycler FastStart DNA Master PLUS SYBR Green I kit (Roche), according to the manufacturer's recommendations. The level of IFNβ was normalized to that of actin based on the second derivative maximum method (Roche). Melting curves were used to verify the specificity of PCR products.

### Antiviral activity of conditioned medium

Conditioned medium was harvested and two-fold serial diluted with fresh medium. 1×10^5^ Vero cells were cultured with the diluted medium for 18 h in a 24-well plate and then infected with dSinF-Luc/2A sindbis virus (500 pfu/well). The cell lysates were harvested for luciferase activity assay (Promega) at 24 h p.i. The conditioned medium collected from DEN-2-infected cells was UV-inactivated [11] before serial dilution.

### Immunofluorescence assay

For IRF3 nuclear translocation assay, cells were fixed with 4% paraformaldehyde in PBS and then permeabilized by 0.5% TritonX-100. After blocking with skim milk in PBS with 0.1% Tween-20 (PBS-T), antibodies against IRF3 (1∶200; Santa Cruz Biotechnology) were added overnight. Biotin-conjugated goat anti-rabbit antibody and Cy3-conjugated streptavidin (1∶1000; Jackson ImmunoResearch) was added sequentially on the next day for 1 h at room temperature. Primary antibodies against JEV and DEN NS1 and NS3 (1∶1000) and goat anti-mouse Alexa Fluor-488-conjugated secondary antibody (1∶1000; Molecular Probes) were used for 1 h at room temperature. Cells were examined and photographed by use of an inverted fluorescent microscope. For the samples examined under a fluorescence laser scanning confocal microscope (FV1000, Olympus), cells were seeded in μ-Slides chamber slides (ibidi) overnight before treatments.

### Western blot analysis

Cells were lysed with RIPA buffer (10 mM Tris, pH 7.5, 5 mM EDTA, 150 mM NaCl, 0.1% SDS, 1% TritonX-100, 1% sodium deoxycholate) containing a cocktail of protease and phosphatase inhibitors (Roche). Protein samples were separated by SDS-PAGE and transferred to a nitrocellulose membrane (Hybond-C Super, Amersham). The nonspecific antibody binding sites were blocked with skim milk in PBS-T and then reacted with the primary antibodies: anti-phospho-IRF3 (S396) (1∶1000; Cell Signaling), anti-IRF3 (1∶1000; Santa Cruz Biotechnology), anti-actin (1∶10000; Chemicon), anti-TMEM173 (1∶2000; Novus), anti-RIG-I (1∶1000; Cell Signaling), anti-MAVS (1∶1,000; Axxora), anti-HA (1∶2000; Covance), anti-V5 (1∶5000; Sigma-Aldrich), and anti-Flag M2 (1∶5000; Sigma-Aldrich). Blots were treated with horseradish peroxidase-conjugated secondary antibody (1∶2500; Jackson ImmunoResearch), and signals were detected by enhanced chemiluminescence (ECL, Pierce). For native PAGE, sample preparation and electrophoresis in the presence of deoxycholate (DOC) were performed as previously described [11]. Briefly, cells were lyzed in protein lysis buffer (50 mM Tris-HCl [pH 7.5], 150 mM NaCl, 1 mM EDTA, 1% NP-40) containing a cocktail of protease inhibitors. Cell lysates (10 µg) in native sample buffer (62.5 mM Tris–HCl [pH 6.8], 15% glycerol, and 1% DOC) were separated by 7.5% PAGE without SDS for 60 min at 25 mA. The electrophoresis buffer contained 25 mM Tris-HCl and 192 mM glycine (pH 8.4) with and without 1% DOC in the cathode and anode chambers, respectively.

### Co-immunoprecipitation

Cells were lysed with protein lysis buffer containing a cocktail of protease and phosphatase inhibitors (Roche). Cell lysates were immunoprecipitated with mouse anti-V5 antibodies (1∶1000; Sigma-Aldrich) or mouse anti-Flag beads (Sigma-Aldrich) overnight at 4°C. The immunocomplex was captured by use of protein G-coated beads (GE Healthcare) at 4°C for 2 h, then precipitates were washed with protein lysis buffer and resuspended in sample buffer with 2-mercaptoethanol. For immunoprecipitation of endogenous MITA, cells were lysed with protein lysis buffer plus 1% TritonX-100. The immunoprecipitated samples were examined by Western blot analysis.

### Statistical analysis

Data are presented as mean ± standard deviation (SD). The results of the indicated groups were compared by two-tailed Student's *t* test.

## Supporting Information

Figure S1 **Dengue NS2B3 suppresses JEV-induced IRF3 phosphorylation.** A549 cells stably transduced with control GFP or DEN-2 NS2B3 were mock-infected (lanes 1–6) or infected with JEV (MOI 5, lanes 7–12) for 16, 24, and 36 h. Immunoblotting was performed with antibodies against pIRF3, IRF3, JEV NS1, GFP, DEN NS3, and actin as indicated.
(TIFF)

Figure S2 **JEV replication level was similar between A549 cells stably expressing GFP or DNS2B3.** Culture supernatants of JEV-infected (MOI 5 for 24 h) A549 stable cells with GFP or dengue NS2B3 (WT or S135A) were analyzed by plaque forming assays for JEV titration. Data are expressed as mean and SD (*n = 3* per group), and were compared by two-tailed Student's *t* test. NS: not significant.
(TIFF)

Figure S3 **Dengue protease downregulates the IFNβ promoter activation triggered by MITA.** A549 cells were cotransfected with p125-Luc (0.15 µg), IRF-3/pCR3.1 (0.15 µg), pRL-TK (0.05 µg), DNS2B3 (WT or S135A, 0.6 µg) and MITA (0.3 µg) for 24 h. GFP transfection was used as a negative control. The cell lysates were harvested and analyzed by dual-luciferase assay as described in Figure 2.
(TIFF)

Figure S4 **Vip-Luc and p125-Luc triggered by TBK1 were not affected by dengue protease.** A549 cells were cotransfected with either reporter (Vip-Luc or p125-Luc; 0.15 µg), IRF-3/pCR3.1 (0.15 µg), pRL-TK (0.05 µg), DNS2B3 (0.35 µg) and TBK1 (0.3 µg) for 24 h. GFP transfection was used as a negative control. The cell lysates were harvested and analyzed by dual-luciferase assay as described in Figure 2.
(TIFF)

Figure S5 **Dengue protease reduced MITA-triggered antiviral activity against VSV.** Vero cells were pretreated with 2-fold serial diluted medium derived from A549 cells cotransfected with DNS2B3 (WT or S135A) plus MITA or GFP control as indicated. The conditioned Vero cells were infected with VSV (25 pfu/well) for 2 days, and adherent cells were stained by crystal violet.
(TIFF)

Figure S6 **DEN-2 infection benefits replication of IFN-sensitive sindbis virus.** (A) Vero cells were treated with various doses of IFNα-2a for 18 h and then infected with recombinant sindbis virus dSinF-Luc/2A for 24 h. Cell lysates were harvested for luciferase activity assay. (B) A549 cells were infected with DEN-2 (MOI 5) for 10 h and then superinfected with dSinF-Luc/2A (MOI 10) for 20 h. Cell lysates were harvested for luciferase activity assay. Data are expressed as mean and SD (*n = 3* per group), and were compared by two-tailed Student's *t* test.
(TIFF)

Figure S7 **Mutation analysis of MITA.** (A) Schematic diagram of MITA/MPYS constructs with mutation sequences. (B) Immunoblotting analysis of A549 cells cotransfected with dengue NS2B3 plus the indicated MITA–mutation constructs. (C) Dual-luciferase assay of A549 cells cotransfected with Vip-Luc, IRF3/pCR3.1, pRL-TK, and the indicated MITA constructs for 24 h as described in Figure 2. (D) Sequences of primers used for MITA/MPYS mutagenesis.
(TIFF)

Figure S8 **DEN protease interacts with MITA but not much with MPYS.** IP-western analysis of A549 cells cotransfected with S135A-mutated dengue NS2B3 plus V5-tagged MITA (WT or IHCM-mutant) or MPYS (WT or LRRG-mutant) for 24 h with the indicated antibodies.
(TIFF)

Figure S9 **Activation of IRF3 in stable cell lines expressing MITA or MPYS upon dsDNA stimulation.** Western blot analysis of pIRF3, IRF3, and actin in A549 stable cell lines overexpressing GFP, MITA, or MPYS transfected with poly(dA:dT) (0.5 or 1 µg) at 6 h post transfection. The band density was quantified with ImageJ and the relative ratios of pIRF3 to IRF3 are shown.
(TIFF)

Figure S10 **DEN infection of stable cell lines overexpressing cleaved-MITA mimics.** A549 stably overexpressing GFP, MITA-N, or MITA-C were infected with DEN-2 (MOI 10) and harvested at the indicated time points post infection. Samples were analyzed by immunoblotting analysis with specific antibodies as indicated.
(TIFF)

Figure S11 **DEN-2 viral production levels in A549 cells with MITA knockdown.** A549 cells stably expressing shRNA targeting *LacZ* or *MITA* were infected with DEN-2 (MOI 0.1 or 10). The culture supernatants were collected for DEN-2 titration at 42 h p.i. by plaque forming assays.
(TIFF)

Figure S12 **MPYS forms cytoplasmic punctate structures with poly(dA:dT) but not poly(I:C) stimulation.** A549 cells with MPYS stable expression were treated with poly(I:C) or poly(dA:dT) (0.5 µg/ml) for 4 h. The cellular distribution of MPYS was revealed by antibody against V5-tag (red). Nuclei stained with DAPI (blue).
(TIFF)

Table S1 **Reduction of IRF3 nucleus translocation by dengue protease.** The raw data collected for calculating the nucleus translocation rate shown in Figure 1B.
(TIFF)
