# Supplementary figures and images for: Dengue Virus Targets the Adaptor Protein MITA to Subvert Host Innate Immunity
Source: PLoS Pathog. 2012 Jun 28;8(6):e1002780. doi: 10.1371/journal.ppat.1002780 (PMC3386177; doi:10.1371/journal.ppat.1002780)

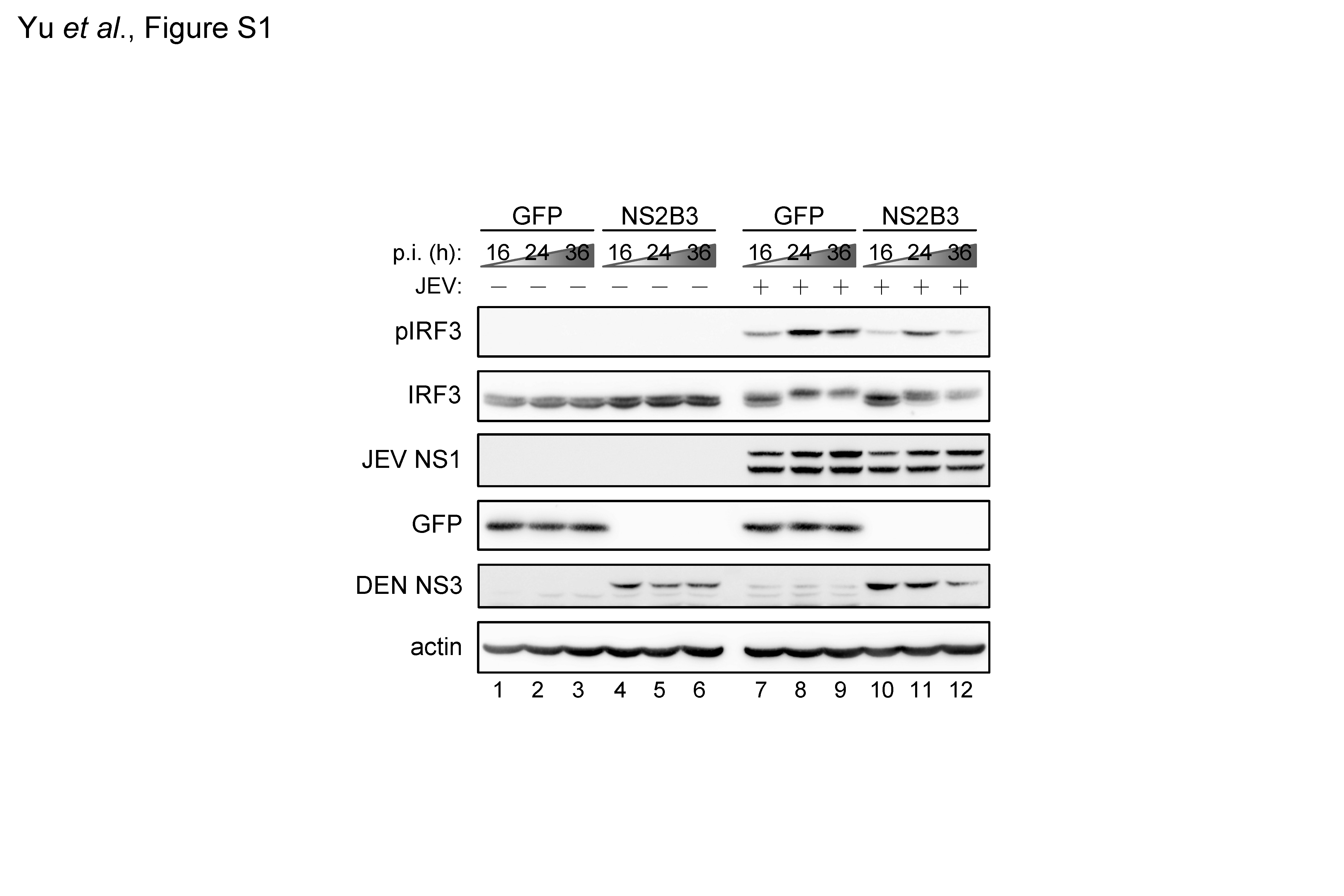

Supplement: Figure S1 — Dengue NS2B3 suppresses JEV-induced IRF3 phosphorylation. A549 cells stably transduced with control GFP or DEN-2 NS2B3 were mock-infected (lanes 1–6) or infected with JEV (MOI 5, lanes 7–12) for 16, 24, and 36 h. Immunoblotting was performed with antibodies against pIRF3, IRF3, JEV NS1, GFP, DEN NS3, and actin as indicated. (TIFF) [file ppat.1002780.s001.tiff]

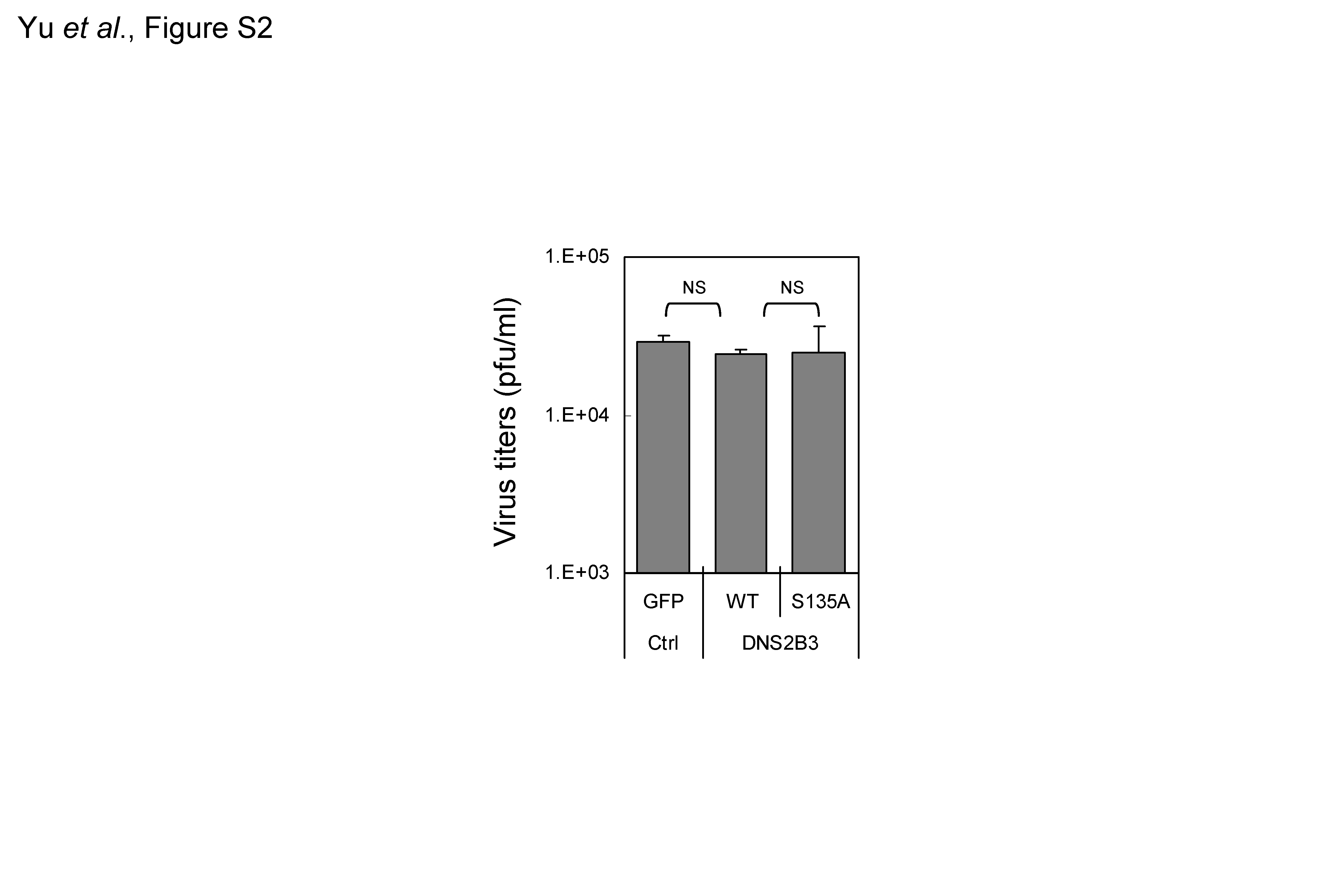

Supplement: Figure S2 — JEV replication level was similar between A549 cells stably expressing GFP or DNS2B3. Culture supernatants of JEV-infected (MOI 5 for 24 h) A549 stable cells with GFP or dengue NS2B3 (WT or S135A) were analyzed by plaque forming assays for JEV titration. Data are expressed as mean and SD (n = 3 per group), and were compared by two-tailed Student's t test. NS: not significant. (TIFF) [file ppat.1002780.s002.tiff]

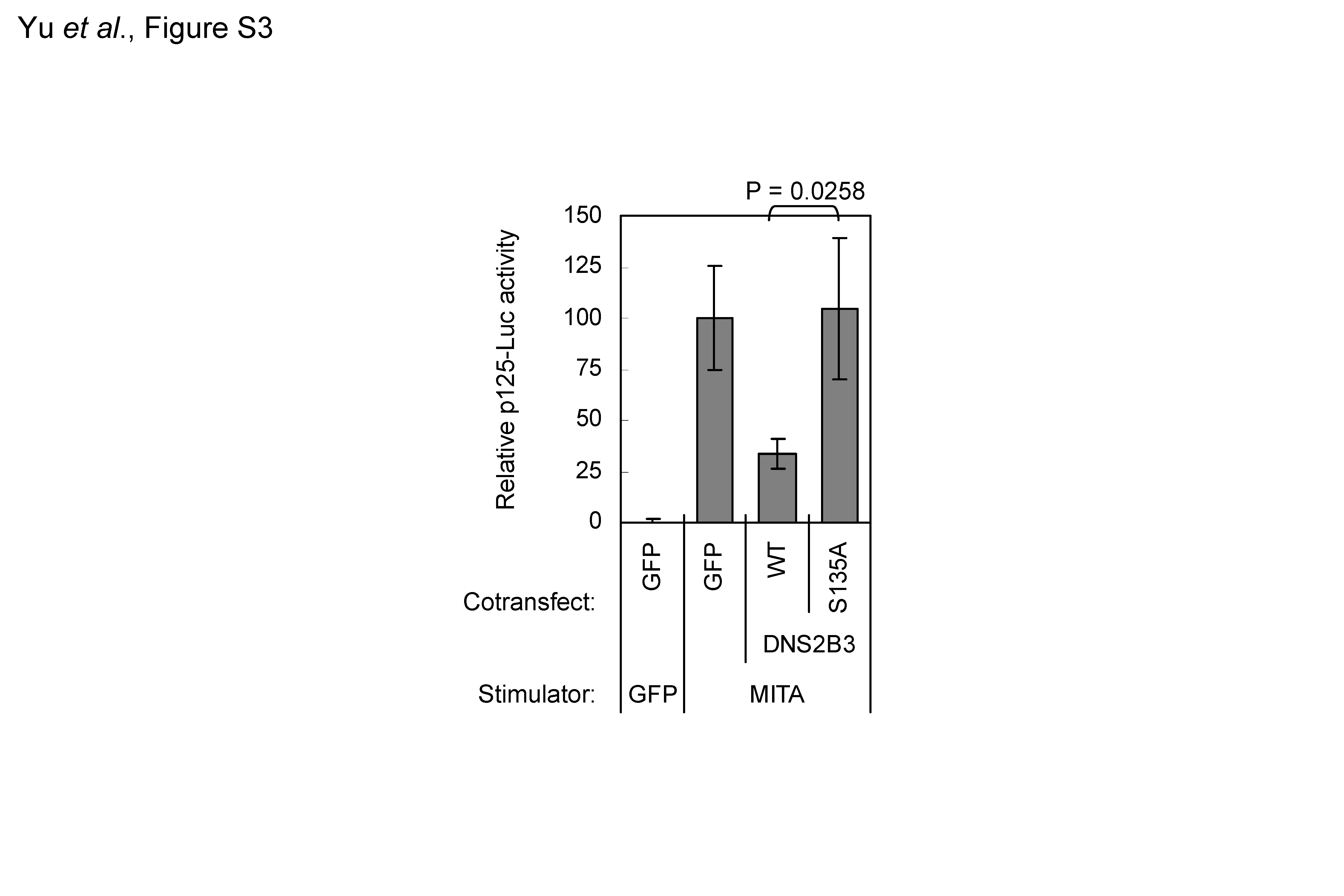

Supplement: Figure S3 — Dengue protease downregulates the IFNβ promoter activation triggered by MITA. A549 cells were cotransfected with p125-Luc (0.15 µg), IRF-3/pCR3.1 (0.15 µg), pRL-TK (0.05 µg), DNS2B3 (WT or S135A, 0.6 µg) and MITA (0.3 µg) for 24 h. GFP transfection was used as a negative control. The cell lysates were harvested and analyzed by dual-luciferase assay as described in Figure 2. (TIFF) [file ppat.1002780.s003.tiff]

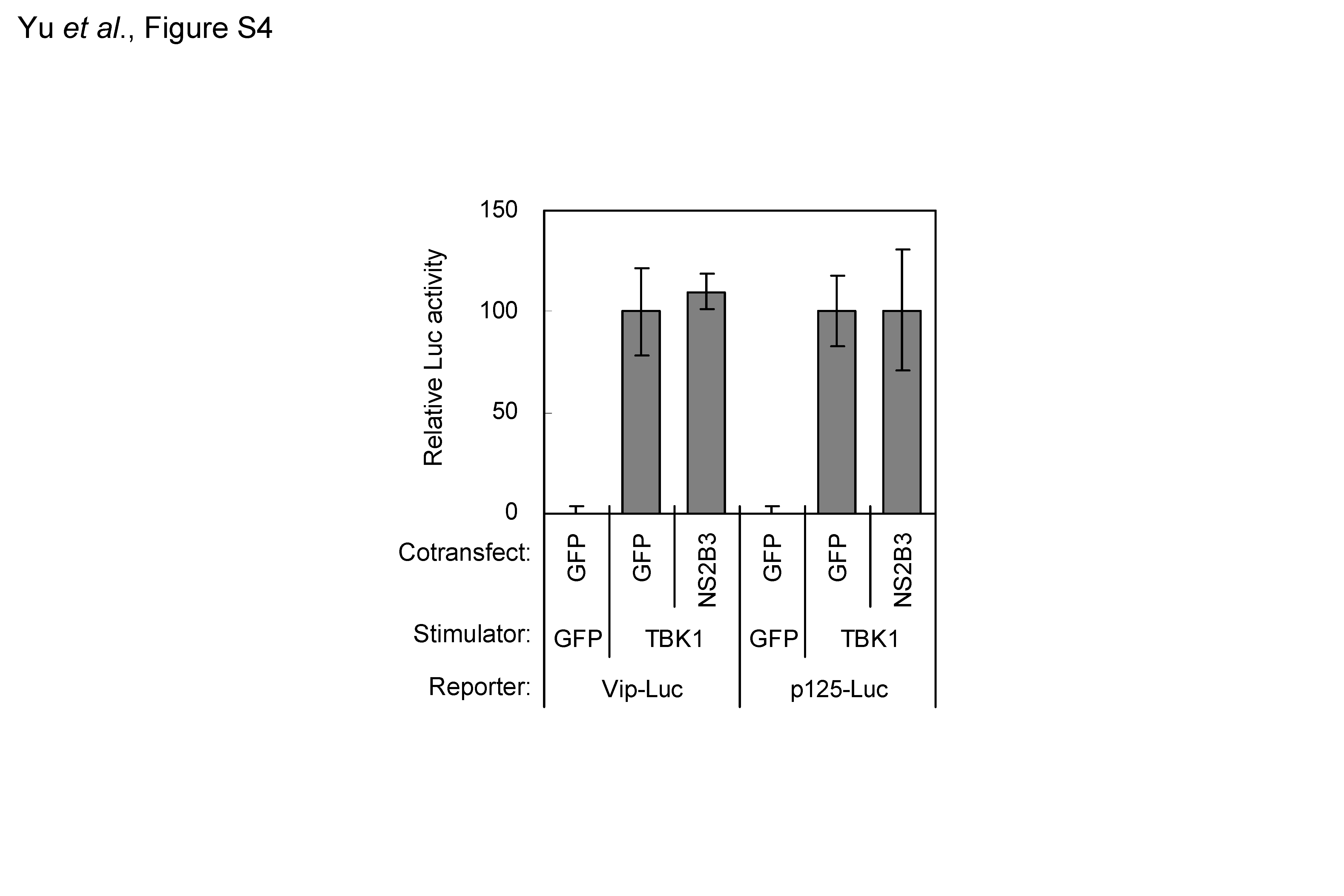

Supplement: Figure S4 — Vip-Luc and p125-Luc triggered by TBK1 were not affected by dengue protease. A549 cells were cotransfected with either reporter (Vip-Luc or p125-Luc; 0.15 µg), IRF-3/pCR3.1 (0.15 µg), pRL-TK (0.05 µg), DNS2B3 (0.35 µg) and TBK1 (0.3 µg) for 24 h. GFP transfection was used as a negative control. The cell lysates were harvested and analyzed by dual-luciferase assay as described in Figure 2. (TIFF) [file ppat.1002780.s004.tiff]

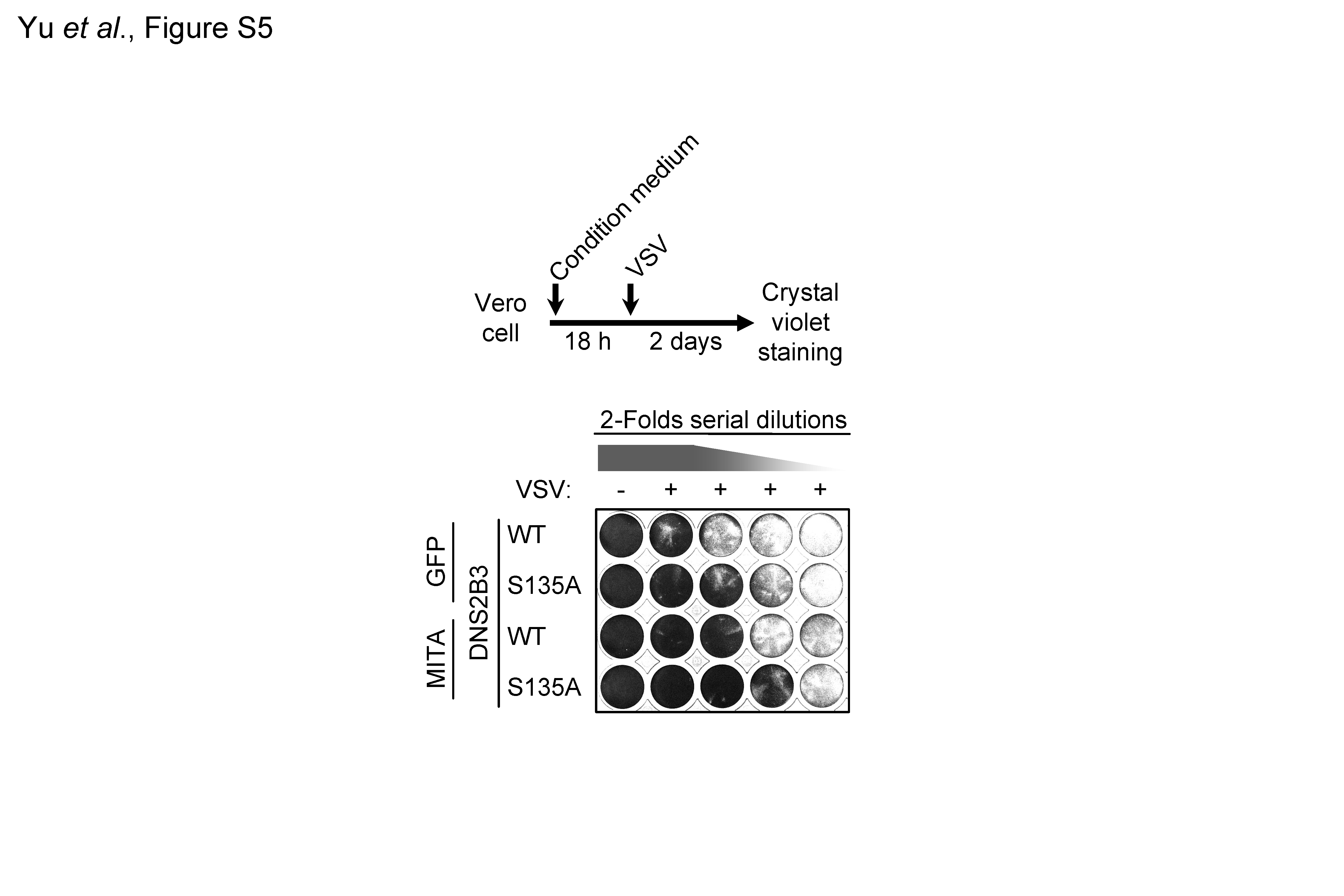

Supplement: Figure S5 — Dengue protease reduced MITA-triggered antiviral activity against VSV. Vero cells were pretreated with 2-fold serial diluted medium derived from A549 cells cotransfected with DNS2B3 (WT or S135A) plus MITA or GFP control as indicated. The conditioned Vero cells were infected with VSV (25 pfu/well) for 2 days, and adherent cells were stained by crystal violet. (TIFF) [file ppat.1002780.s005.tiff]

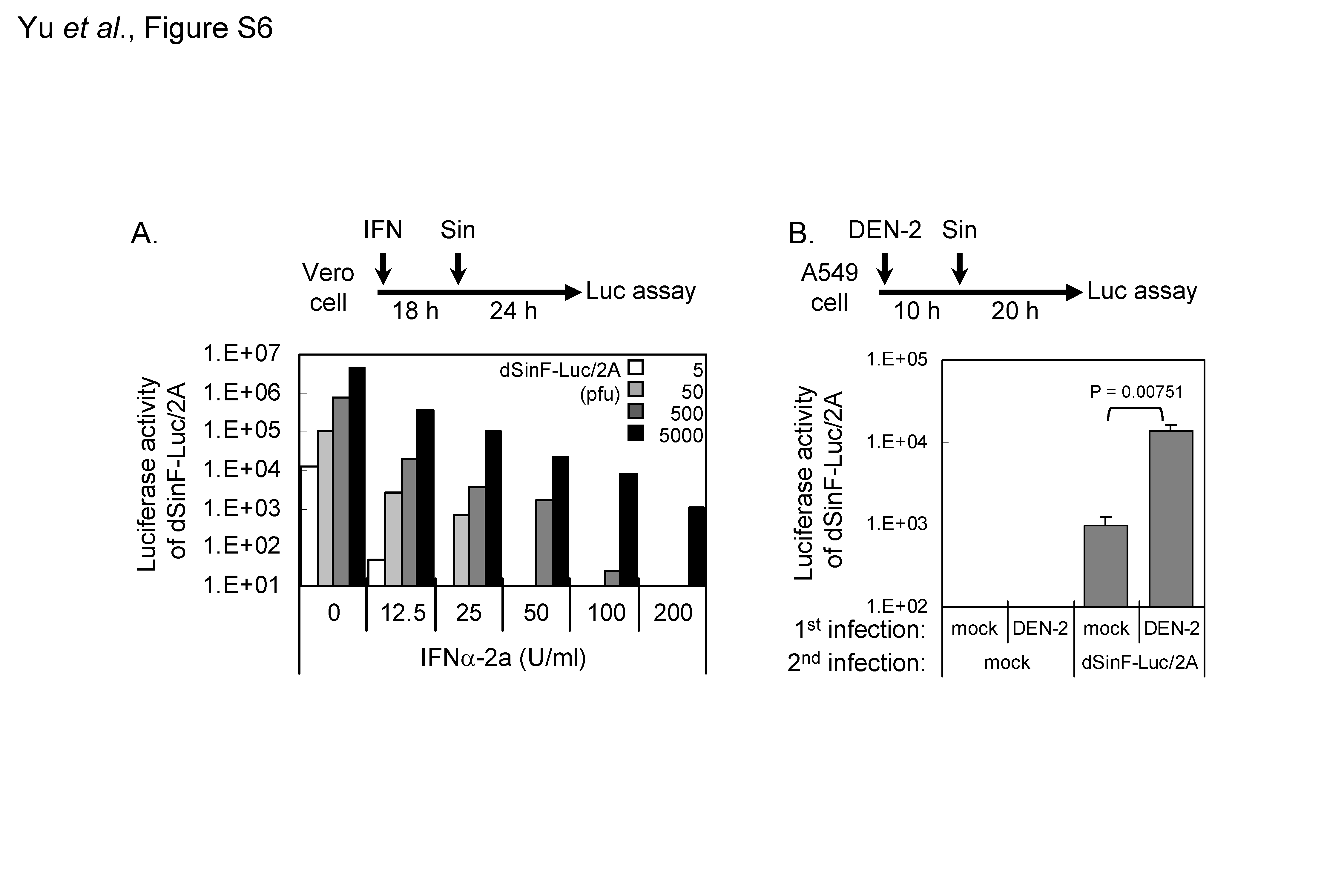

Supplement: Figure S6 — DEN-2 infection benefits replication of IFN-sensitive sindbis virus. (A) Vero cells were treated with various doses of IFNα-2a for 18 h and then infected with recombinant sindbis virus dSinF-Luc/2A for 24 h. Cell lysates were harvested for luciferase activity assay. (B) A549 cells were infected with DEN-2 (MOI 5) for 10 h and then superinfected with dSinF-Luc/2A (MOI 10) for 20 h. Cell lysates were harvested for luciferase activity assay. Data are expressed as mean and SD (n = 3 per group), and were compared by two-tailed Student's t test. (TIFF) [file ppat.1002780.s006.tiff]

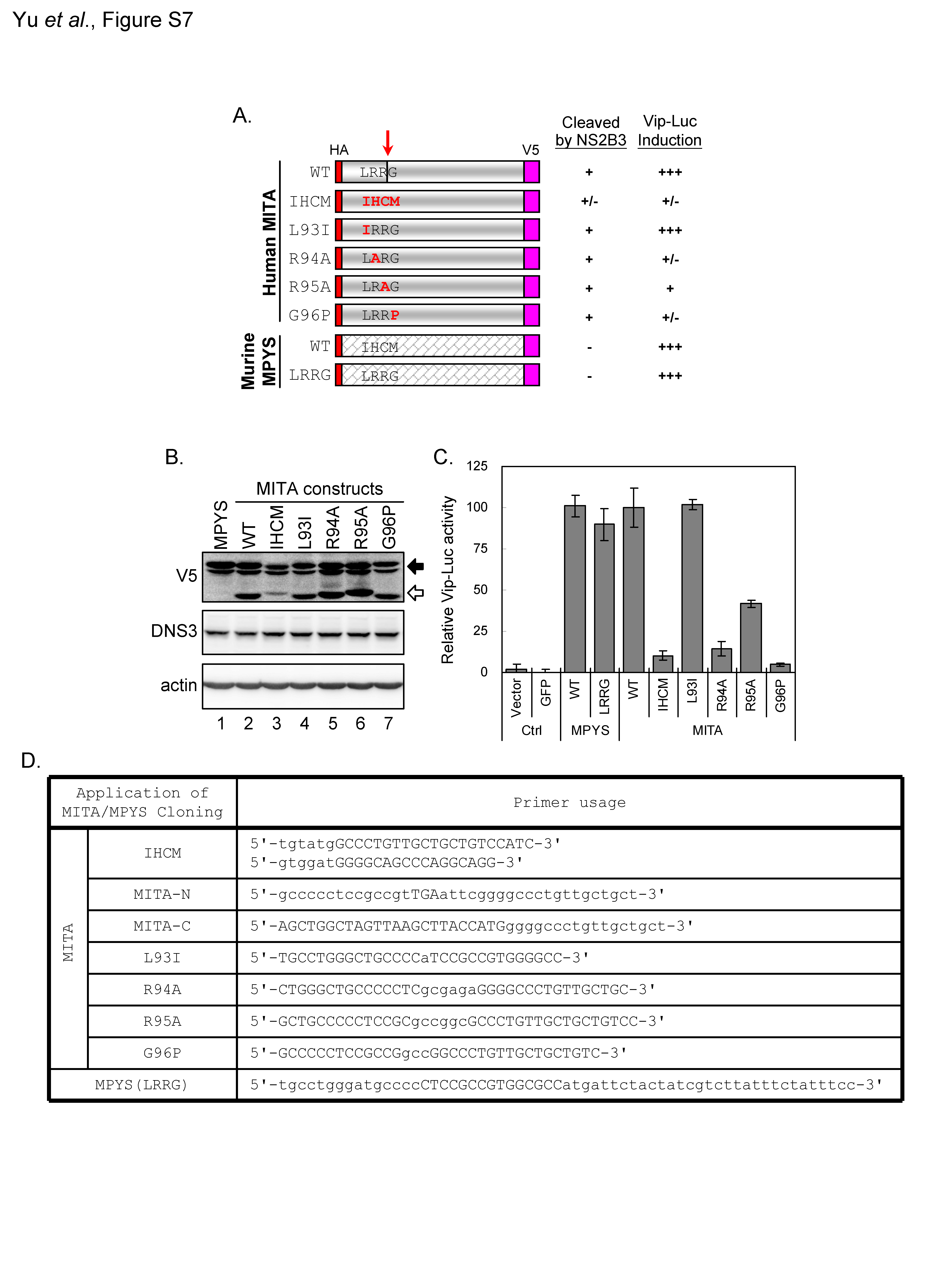

Supplement: Figure S7 — Mutation analysis of MITA. (A) Schematic diagram of MITA/MPYS constructs with mutation sequences. (B) Immunoblotting analysis of A549 cells cotransfected with dengue NS2B3 plus the indicated MITA–mutation constructs. (C) Dual-luciferase assay of A549 cells cotransfected with Vip-Luc, IRF3/pCR3.1, pRL-TK, and the indicated MITA constructs for 24 h as described in Figure 2. (D) Sequences of primers used for MITA/MPYS mutagenesis. (TIFF) [file ppat.1002780.s007.tiff]

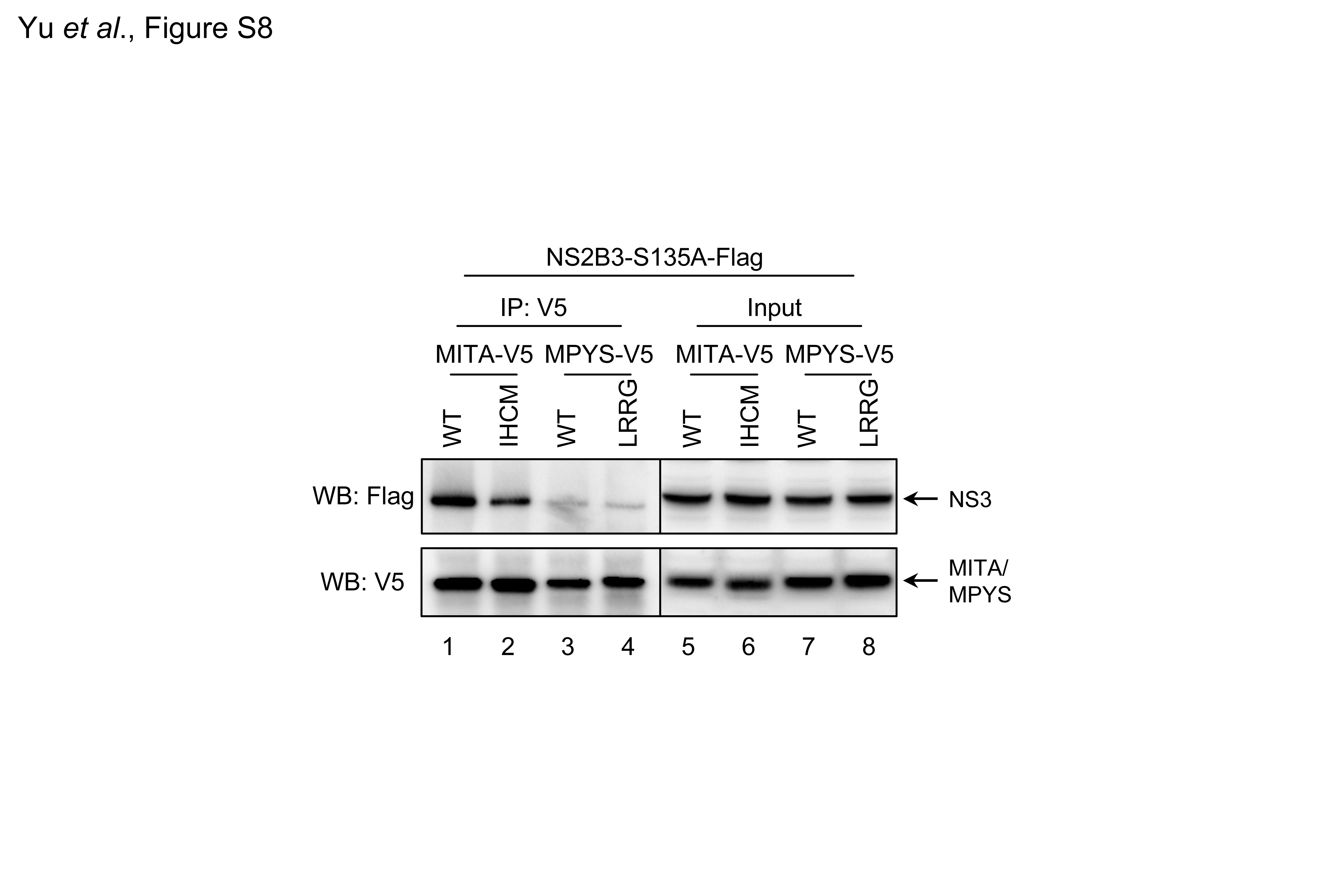

Supplement: Figure S8 — DEN protease interacts with MITA but not much with MPYS. IP-western analysis of A549 cells cotransfected with S135A-mutated dengue NS2B3 plus V5-tagged MITA (WT or IHCM-mutant) or MPYS (WT or LRRG-mutant) for 24 h with the indicated antibodies. (TIFF) [file ppat.1002780.s008.tiff]

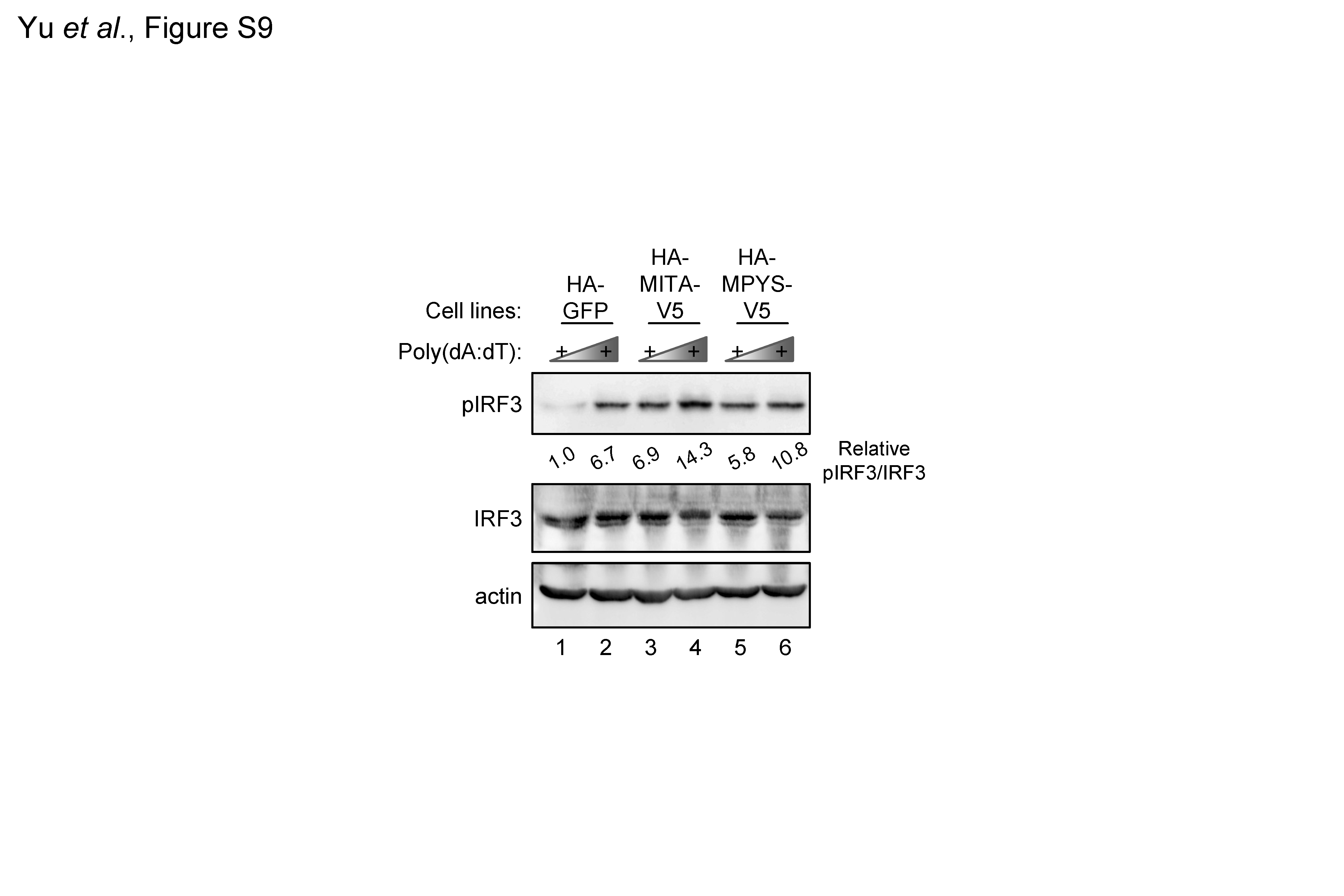

Supplement: Figure S9 — Activation of IRF3 in stable cell lines expressing MITA or MPYS upon dsDNA stimulation. Western blot analysis of pIRF3, IRF3, and actin in A549 stable cell lines overexpressing GFP, MITA, or MPYS transfected with poly(dA:dT) (0.5 or 1 µg) at 6 h post transfection. The band density was quantified with ImageJ and the relative ratios of pIRF3 to IRF3 are shown. (TIFF) [file ppat.1002780.s009.tiff]

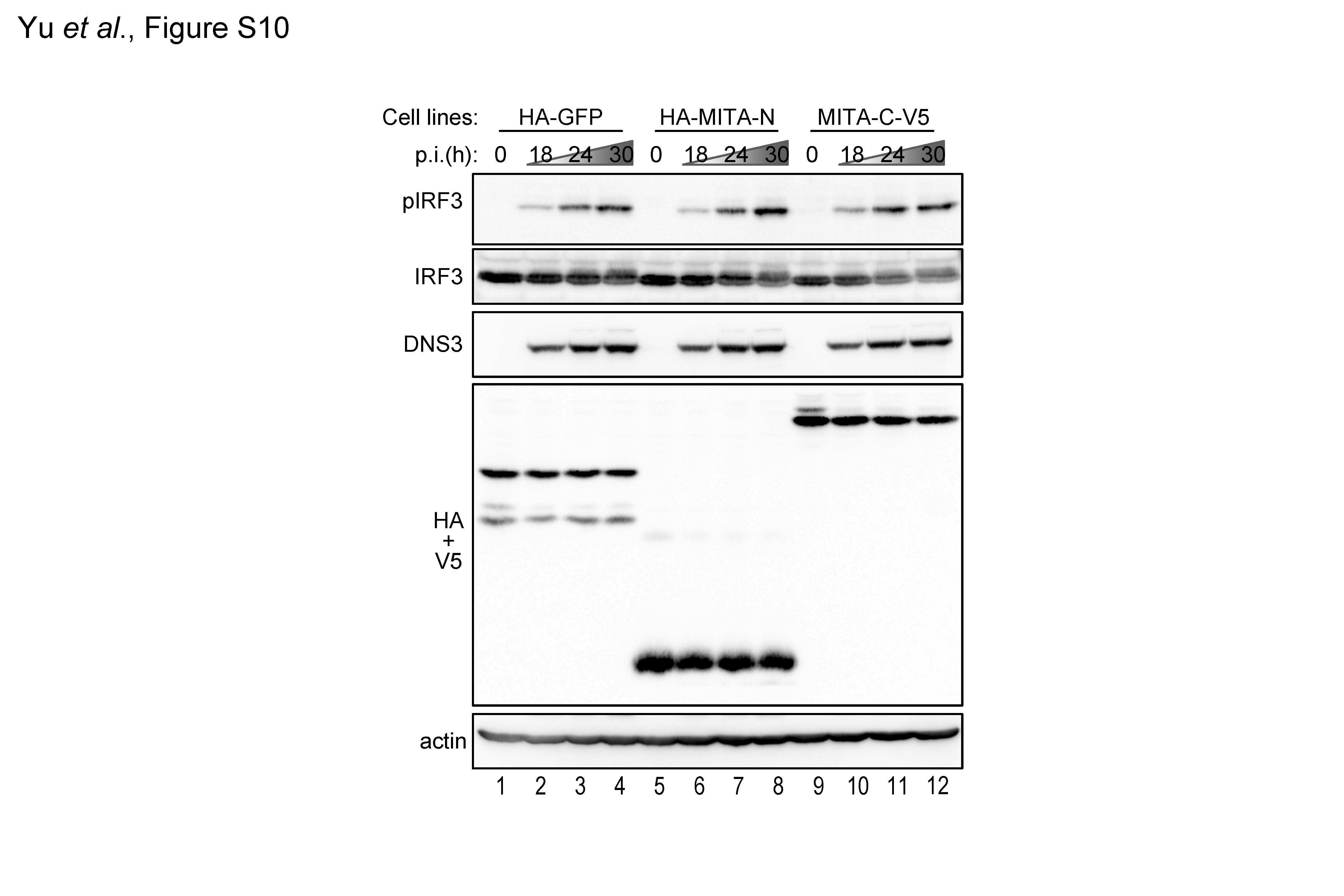

Supplement: Figure S10 — DEN infection of stable cell lines overexpressing cleaved-MITA mimics. A549 stably overexpressing GFP, MITA-N, or MITA-C were infected with DEN-2 (MOI 10) and harvested at the indicated time points post infection. Samples were analyzed by immunoblotting analysis with specific antibodies as indicated. (TIFF) [file ppat.1002780.s010.tiff]

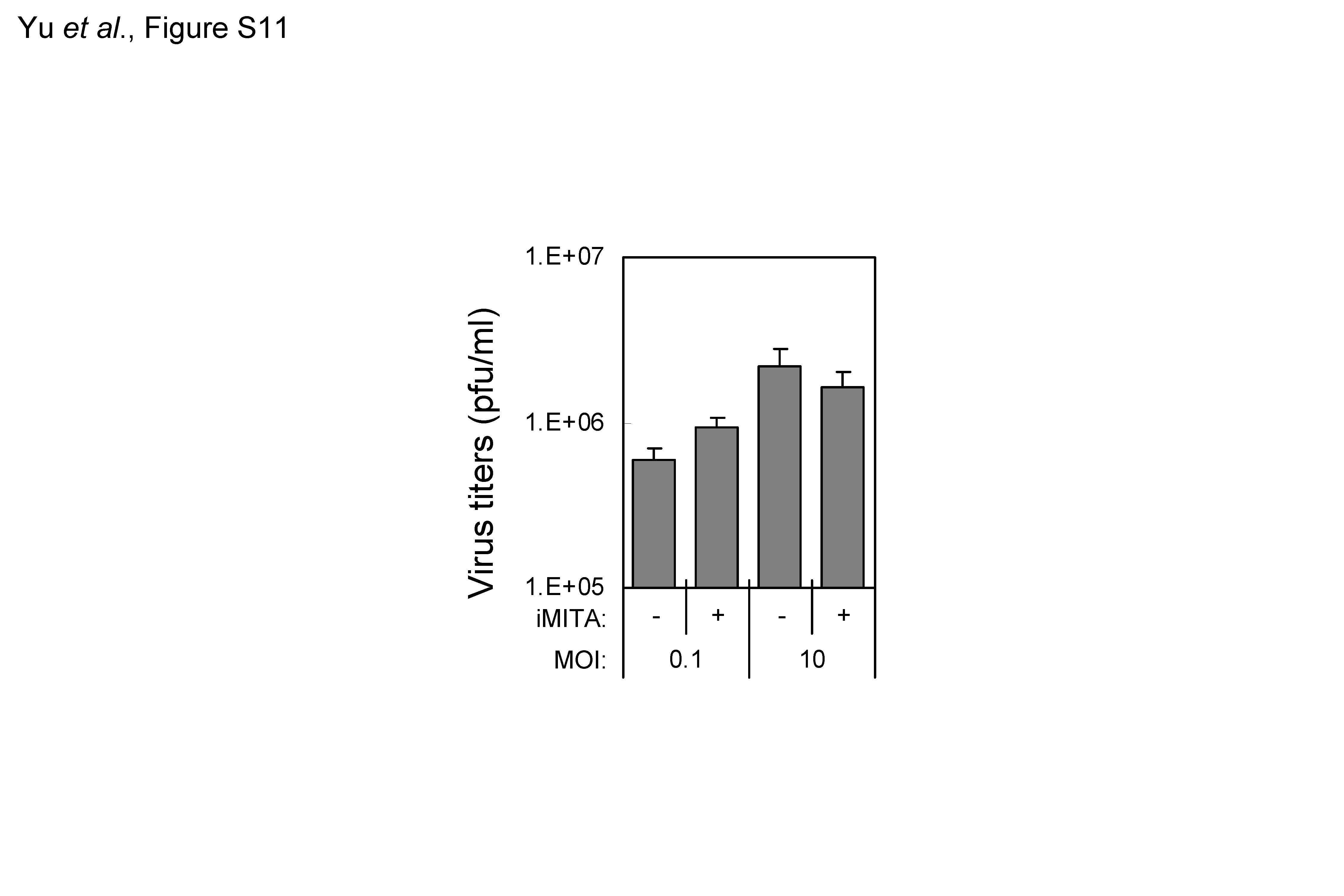

Supplement: Figure S11 — DEN-2 viral production levels in A549 cells with MITA knockdown. A549 cells stably expressing shRNA targeting LacZ or MITA were infected with DEN-2 (MOI 0.1 or 10). The culture supernatants were collected for DEN-2 titration at 42 h p.i. by plaque forming assays. (TIFF) [file ppat.1002780.s011.tiff]

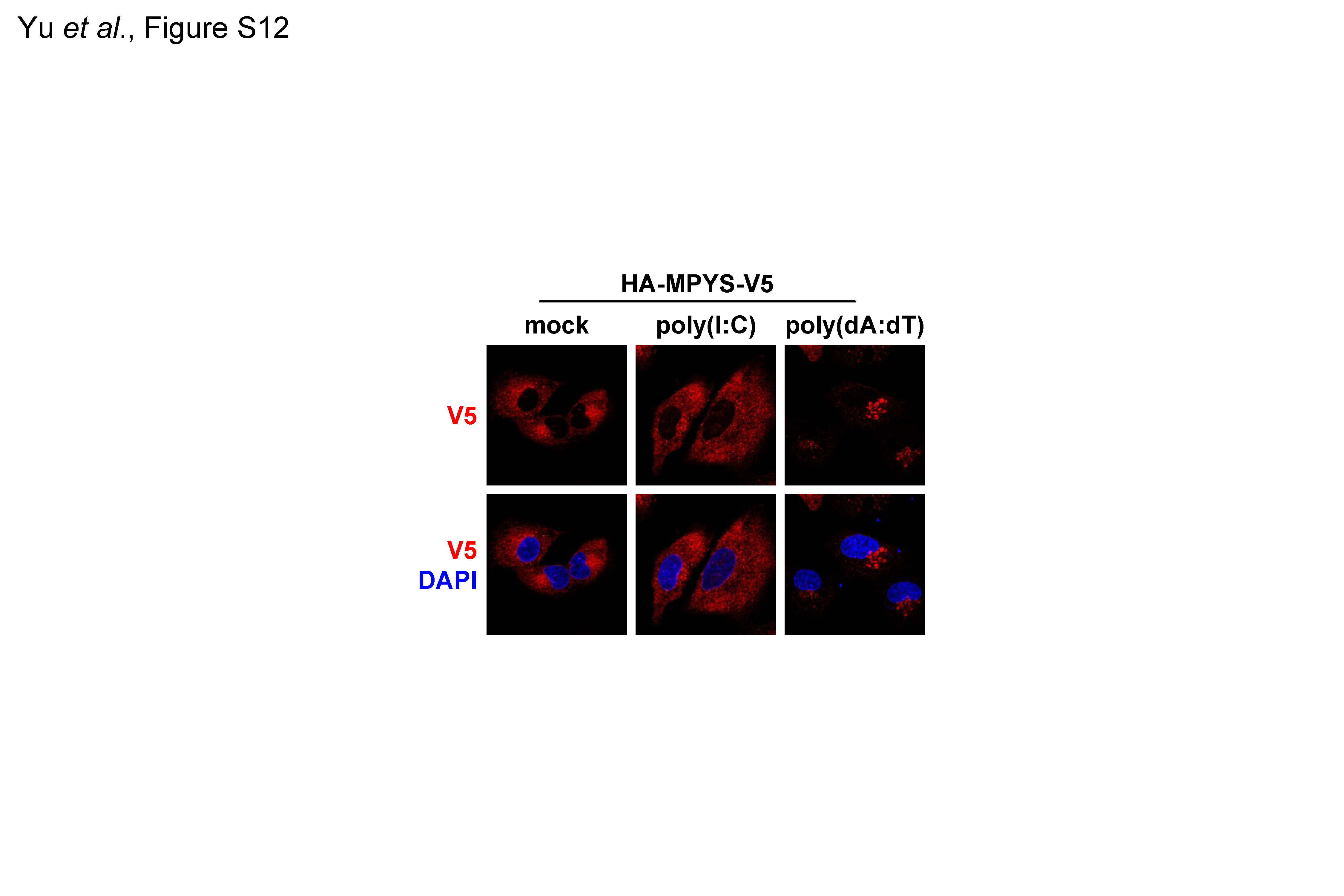

Supplement: Figure S12 — MPYS forms cytoplasmic punctate structures with poly(dA:dT) but not poly(I:C) stimulation. A549 cells with MPYS stable expression were treated with poly(I:C) or poly(dA:dT) (0.5 µg/ml) for 4 h. The cellular distribution of MPYS was revealed by antibody against V5-tag (red). Nuclei stained with DAPI (blue). (TIFF) [file ppat.1002780.s012.tiff]
